# The factors that govern the allosteric chemical sensing of polythiophene chemosensors: scope and limitation toward signal-amplification sensing†

**DOI:** 10.1039/d1ra05795b

**Published:** 2021-09-14

**Authors:** Tomoaki Tsuchiya, Hiroaki Mizuno, Gaku Fukuhara

**Affiliations:** Department of Chemistry, Tokyo Institute of Technology 2-12-1 Ookayama Meguro-ku Tokyo 152-8551 Japan gaku@chem.titech.ac.jp; JST, PRESTO 4-1-8 Honcho Kawaguchi Saitama 332-0012 Japan

## Abstract

The newly designed polythiophene chemosensors (PT1 and PT2) were synthesized *via* the Suzuki–Miyaura polymerization with appropriate yields. The photophysical properties of PTs thus obtained were examined by means of UV/vis, fluorescence, excitation spectroscopy, and time-correlated single-photon-counting method. The π–π* transitions around 400–600 nm and the emissions in the range of 400–650 nm were observed. The binding behavior of PTs was also investigated upon the interaction of tetrabutylammonium or tetrabutylphosphonium isophthalate, affording the binding constants (*K*) of 5790–8310 M^−1^, which were quite smaller than those observed in the corresponding repeating unit. The comprehensive analyses of the UV/vis data and theoretical calculation supports revealed the origins of scope and limitation toward signal-amplification sensing. The present results obtained herein will guide the development of new amplification chemosensors.

## Abstract 2

The allosteric sensing of target guest molecules was drastically inhibited by introducing thiophene spacers in the polythiophene backbone, which is caused by the conformational relaxation.

## Graphical abstract

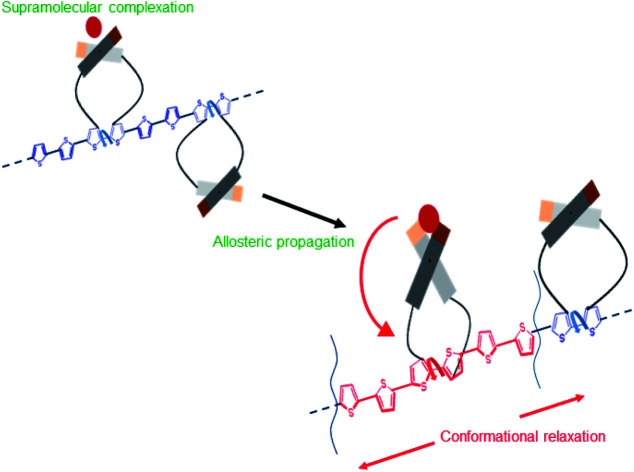

## Introduction

Chemical sensing of target molecules with chemosensors has become a recent trend in the fields of chemistry due to some applications in the precise detection of hazardous explosives and toxic vapors (VOCs)^1–3^ and biomarkers relating to serious diseases or cancers.^4–6^ The previous chemosensors have been achieved based on the “lock-and-key” model, which was proposed as activations of enzymes by Emil Fischer since the late 1800's,^7^ and thus, many sophisticated reports have been exemplified so far.^8–19^ Nevertheless, unluckily, such a model is not as almighty since target analytes have become complicated structurally and conformationally, *e.g.*, peptides, proteins, and carbohydrates.^20–24^ This current research implies the necessity of an alternative to the conventional lock-and-key principle-based chemosensor construction.^25^

**Fig. 1:**
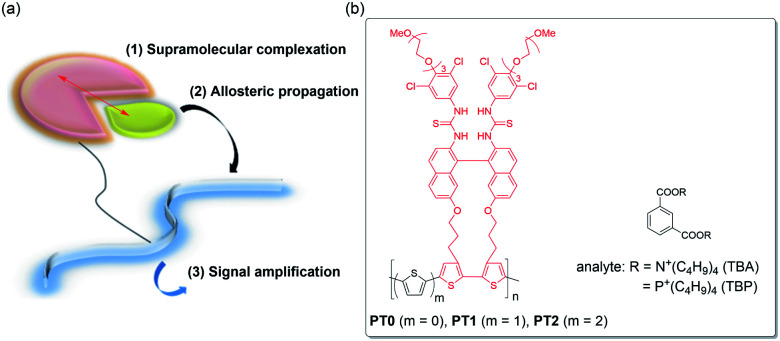
(a) The basic idea of supramolecular allosteric signal-amplification sensing (SASS). (b) Chemical structures of polythiophene chemosensors (PT0, PT1, and PT2) and analytes (TBA and TBP), in which the triad monomer (TM) unit is highlighted in red.

A smarter sensing method seems to be a signal amplification, *e.g.*, polymerase chain reaction (PCR)^26^ and enzyme-linked immunosorbent assay (ELISA),^27^ the former of which has been well-known owing to the Covid-19 disaster in recent years. Recently, as a signal-amplification reporter, polymer chains turn out to be good candidates. In a pioneering work in 1995, Swager *et al.* demonstrated the prominently increased sensitivity of a target molecule using conjugated polymers, which is called as the molecular wire approach.^28,29^ Indeed, a crown ether cavity was incorporated in the conjugated polymer chains, *i.e.*, a type of cyclophane receptor repeating unit, causing pseudorotaxane complexation upon the interaction of the target paraquat guest with the enhancement in sensitivity by a factor of 64 compared to the corresponding repeating unit. The amplification by the molecular wire effect is ascribable to a facile energy migration onto the conjugated polymer chains. On the other hand, we proposed a novel amplification method as “supramolecular allosteric signal-amplification sensing” (SASS) in 2015, which is shown in Fig. 1a.^21–24^ The SASS approach consists of the following three steps: (1) supramolecular complexation of a target analyte with a recognition site in a chemosensor skeleton, (2) allosteric propagation of binding information upon the complexation of the analyte through a polymer chain, and (3) signal amplification caused by an enhanced complexation stability of the analyte into an induced-fit site. The key mechanisms of SASS are assignable to the synchronized conformational changes of polymer backbones and the subsequent allosteric complexation of an analyte, which is quite different from the previous molecular wire approach relating to the complex excited-state behavior. Therefore, the SASS strategy appears to be simpler as an idea, but the molecular design of polymers may be rather critical.

Very recently, to further develop a new SASS system, we synthesized functional polythiophene (PT0), including the triad monomer unit (TM) composed of bisthiourea as the molecular recognition tweezer moiety, binaphthyl as the signal transducer skeleton, and bithiophene as a part of the signal reporter unit, and then, examined the amplification sensing behavior of phthalic acids and phthalates (see the structures in Fig. 1b).^30^ In fact, the PT0 sensor showed extremely large binding constants (*K*) enhanced by a factor of 5–15 compared to the TM sensor, for which the applicable *syn*–*anti* conformational changes of the polythiophene (PT) chain are responsible. However, we did not completely understand whether the direct connection between the bithiophene units possessing the molecular recognition moiety is really proper because the synchronized backbone changes are undoubtedly the key point from the viewpoint of the SASS mechanism.

In the present study, to expand the scope of the SASS using the functional TM unit, we want to elaborate on the effects on how to connect TM*via* monothiophene (PT1) or bithiophene spacer (PT2) shown in Fig. 1b. The comprehensive sensing studies on PT1 and PT2 by comparing to those obtained in PT0 enable us to conclude the SASS mechanistic insights into the factors that govern the amplification sensing outcomes. Since the synthetic chemistry of PTs has considerably been established,^31^ the results obtained from this study can easily feedback to the chemistry of PT-based chemosensors.^32,33^

## Experimental

### Materials

All chemicals and solvents were employed as received. Spectroscopic-grade chloroform was used for spectroscopy without further purification. Tetrabutylammonium (TBA) and tetrabutylphosphonium isophthalates (TBP) were prepared according to the appropriate protocol.^34,35^ Concentrations of the synthesized PTs are expressed as monomer unit throughout this work. All spectra were measured in a 1 cm cell in CHCl_3_ at 25 °C unless stated otherwise.

### Instruments

Melting points were measured by a Büchi M-560 apparatus. HRMS spectra were recorded on a Bruker ESI microTOF II instrument. ^1^H, ^13^C, 2D-COSY, and -HMQC NMR spectra (400 or 100 MHz) were measured in CDCl_3_ containing tetramethylsilane (TMS) as an internal standard with an ECX400 NMR spectrometer. UV/vis spectra were measured in a quartz cell (1 cm path length) by recording on a JASCO V-650 spectrometer equipped with an ETCS-761 temperature controller. Fluorescence spectra were measured in the same cell using a JASCO FP-8500 spectrophotometer equipped with an ETC-815 temperature controller. Fluorescence lifetime decays were observed in the same cell by a Hamamatsu Quantaurus-Tau single-photon-counting instrument fitted with an LED excitation light (340 nm). IR spectra were measured on a JASCO FT/IR-4700 spectrometer. The molecular weights of the PTs were determined using polystyrene standards through an analytical GPC with a TOSOH TSKgel α-4000 column (condition: 40 °C, 0.5 mL min^−1^, DMF).

### Synthesis of monomer

#### Synthesis of 3,3′-(5,5′-dibromo-[2,2′-bithiophene]-3,3′-diyl)bis(propan-1-ol)

*n*-BuLi (4.4 mL, 1.6 M in *n*-hexane, 7.04 mmol) was added to a 50 mL round-bottomed flask containing 3-(3-(2-tetrahydropyranyloxy)propyl)thiophene^30^ (1.60 g, 7.09 mmol) dissolved in dry THF (8.5 mL) at 0 °C and the mixture was stirred for 30 min at 0 °C. The mixture was transferred to a two-necked 200 mL round-bottomed flask containing Fe(acac)_3_ (2.49 g, 7.06 mmol) dissolved in dry THF (43 mL), and then the resulting mixture was stirred in reflux overnight. The solution was quenched with water in an ice bath and then filtered while washing the salts with THF. The filtrate was extracted with EtOAc and water. The organic layer obtained was dried over anhydrous MgSO_4_, filtered, and then concentrated under a high vacuum. Flash column chromatography (EtOAc, 1 : 10 EtOAc/*n*-hexane) of the residue afforded the dimerized product as a yellow oil (1.19 g, 2.64 mmol (containing 14% impurities)). The yellow oil (1.19 g, 2.64 mmol) and *n*-bromosuccinimide (0.99 g, 5.58 mmol) in a 50 mL round-bottomed flask were dissolved in THF (4 mL) and acetic acid (4 mL), and the mixture was stirred for 4 h at room temperature. Then, the solution was extracted with diethyl ether and saturated aqueous sodium bicarbonate solution. The organic layer obtained was dried over anhydrous Na_2_SO_4_, filtered, and evaporated under a high vacuum. Flash column chromatography (7 : 3 *n*-hexane/EtOAc) of the residue gave the brominated product as an orange oil (1.30 g, 2.14 mmol (containing 14% impurities)). The orange oil (1.30 g, 2.14 mmol) and pyridinium *p*-toluenesulfate (0.23 g, 0.92 mmol) were dissolved in dry MeOH (9 mL), and the mixture was stirred overnight at 50 °C. Then, the solution was extracted with dichloromethane and water. The organic layer obtained was dried over anhydrous MgSO_4_, filtered, and evaporated under high vacuum. Flash column chromatography (7 : 3–3 : 7 *n*-hexane/EtOAc) of the residue gave the desired product (250 mg, 0.89 mmol) as a white solid in 33% yield, mp: 91–92 °C; HRMS (ESI^+^, TOF): calculated for C_14_H_17_Br_2_O_2_S_2_ [M + H]^+^: 438.9031, found 438.9011; ^1^H NMR (CDCl_3_, 400 MHz, 20 °C) *δ*_H_ 6.80 (s, 2H), 3.68 (q, *J* = 7.3 Hz, 4H), 2.65 (t, *J* = 7.8 Hz, 4H), 1.87 (q, *J* = 7.1 Hz, 4H), 1.52 (br, 2H); ^13^C NMR (CDCl_3_, 100 MHz, 20 °C) *δ*_C_ 141.9, 136.2, 124.4, 108.3, 61.9, 32.3, 25.8.

#### Synthesis of (5,5′-dibromo-[2,2′-bithiophene]-3,3′-diyl)bis(propane-3,1-diyl)bis(4-methylbenzenesulfonate)

3,3′-(5,5′-Dibromo-[2,2′-bithiophene]-3,3′-diyl)bis(propan-1-ol) (279 mg, 0.63 mmol) in a 20 mL round-bottomed flask was dissolved in dry pyridine (6 mL) and stirred for 30 min at 0 °C. Then, toluenesulfonyl chloride (600 mg, 3.15 mmol) was added to the mixture and stirred for 8 h at 0 °C. The reaction mixture was extracted with dichloromethane and 3 M HCl. The organic layer obtained was dried over anhydrous MgSO_4_, filtered, and evaporated under high vacuum. Flash column chromatography (CH_2_Cl_2_) of the residue gave the desired product (323 mg, 0.43 mmol) as a white foamy powder in 68% yield, mp: 114–115 °C; HRMS (ESI^+^, TOF): calculated for C_28_H_28_Br_2_O_6_S_4_Na [M + Na]^+^: 768.9033, found 768.9032; ^1^H NMR (CDCl_3_, 400 MHz, 20 °C) *δ*_H_ 7.80 (d, *J* = 8.4 Hz, 4H), 7.34 (d, *J* = 8.3 Hz, 4H), 6.70 (s, 2H), 4.06 (t, *J* = 6.3 Hz, 4H), 2.60 (t, *J* = 7.7 Hz, 4H), 2.45 (s, 6H), 1.95 (q, *J* = 7.0 Hz, 4H); ^13^C NMR (CDCl_3_, 100 MHz, 20 °C) *δ*_C_ 144.8, 140.6, 136.3, 132.9, 129.9, 127.9, 124.4, 109.0, 69.3, 28.6, 25.4, 21.7.

#### Synthesis of binaphthyl-dibromobithiophene conjugate

(5,5′-Dibromo-[2,2′-bithiophene]-3,3′-diyl)bis(propane-3,1-diyl)bis(4-methylbenzenesulfonate) (323 mg, 0.43 mmol), 2,2′-diamino-[1,1′-binaphthalene]-7,7′-diol^30^ (136 mg, 0.43 mmol), and K_2_CO_3_ (298 mg, 2.16 mmol) in a round-bottomed flask were dissolved in dry DMF (80 mL) and the mixture was stirred for 6 d at 50 °C. The reaction mixture was extracted with dichloromethane and water, and the organic layer was washed several times with water to remove DMF. The organic layer obtained was dried over anhydrous MgSO_4_ to remove the solvent. Flash column chromatography (3 : 2 *n*-hexane/EtOAc) of the residue gave the desired product (95 mg, 0.13 mmol) as a white solid in 30% yield, mp: 126–127 °C; HRMS (ESI^+^, TOF): calculated for C_34_H_28_Br_2_O_2_S_2_Na [M + Na]^+^: 740.9857, found 740.9898; ^1^H NMR (CDCl_3_, 400 MHz, 20 °C) *δ*_H_ 7.72–7.69 (m, 4H), 6.98–6.92 (m, 4H), 6.37 (s, 2H), 6.29 (s, 2H), 3.60–3.43 (m, 4H), 2.82–2.68 (m, 4H), 2.01–1.79 (m, 4H); ^13^C NMR (CDCl_3_, 100 MHz, 20 °C) *δ*_C_ 158.0, 149.3, 143.1, 140.8, 135.1, 129.7, 129.1, 127.2, 123.9, 115.8, 113.8, 112.0, 109.0, 103.2, 63.8, 27.3, 24.6.

#### Synthesis of bisthiourea-binaphthyl-dibromobithiophene conjugate (dibrominated TM)

To a 10 mL flask containing the binaphthyl-dibromobithiophene conjugate (77 mg, 0.11 mmol), 1,3-dichloro-5-isothiocyanato-2-(2-(2-(2-methoxyethoxy)ethoxy)ethoxy)benzene^30^ (162 mg, 0.44 mmol) in dry THF (3 mL) was added and the mixture was stirred for 6 d at room temperature. The solvent was removed under high vacuum. Flash column chromatography (7 : 3 CH_2_Cl_2_/diethyl ether) of the residue gave the desired product as a white powder (98 mg, 0.068 mmol) in 64% yield, mp: 106–107 °C; HRMS (ESI^+^, TOF): calculated for C_62_H_63_Br_2_Cl_4_N_4_O_10_S_4_ [M + H]^+^: 1449.0548, found 1449.6399; ^1^H NMR (CDCl_3_, 400 MHz, 20 °C) *δ*_H_ 7.94–7.89 (m, 4H), 7.59 (d, *J* = 8.9 Hz, 2H), 7.35 (s, 2H), 7.22–7.19 (m, 4H), 6.45 (s, 2H), 6.42 (s, 4H), 6.15 (s, 2H), 4.13 (t, *J* = 5.1 Hz, 4H), 3.86 (t, *J* = 5.1 Hz, 4H), 3.75–3.65 (m, 12H), 3.57–3.54 (m, 4H), 3.37 (s, 6H), 2.75–2.64 (m, 4H), 2.01–1.82 (m, 4H); ^13^C NMR (CDCl_3_, 100 MHz, 20 °C) *δ*_C_ 180.6, 158.0, 151.0, 140.4, 135.7, 134.9, 133.7, 131.5, 130.1, 130.0, 128.6, 128.1, 127.7, 127.3, 126.6, 124.5, 119.1, 109.2, 103.5, 72.6, 71.9, 70.7, 70.6, 70.5, 70.0, 64.5, 59.0, 27.0, 24.8.

### Synthesis of polymers

#### Synthesis of PT1

The dibrominated TM (50 mg, 0.034 mmol), 2,5-bis(4,4,5,5-tetramethyl-1,3,2-dioxaborolan-2-yl)thiophene (12 mg, 0.034 mmol), K_3_PO_4_ (214 mg, 1.01 mmol), and Pd(PPh_3_)_4_ (20 mg, 50 mol%) were dissolved in dry DMF (10 mL) and the solution was stirred for 11 d at 90 °C under N_2_ atmosphere. The solvent was removed under high vacuum. The residue was dissolved in DMF (1 mL), and the solution was slowly poured into *n*-hexane (20 mL). The precipitate obtained was filtered, collected, and dried under high vacuum to give PT1 (15 mg, 0.011 mmol in monomer unit) as an orange solid in 33% yield. ^1^H NMR (CDCl_3_, 400 MHz, 20 °C) *δ*_H_ 8.06–6.04 (br, 22H), 4.33–3.08 (br, 34H), 2.94–2.52 (br, 4H), 2.15–1.65 (br, 4H); ^13^C NMR (CDCl_3_, 400 MHz, 20 °C) *δ*_C_ 157.8, 140.5, 132.3, 131.1, 130.0, 128.9, 119.0, 72.6, 72.0, 70.7, 70.2, 65.7, 59.1, 29.8, 28.5, 25.8; *M*_n_ = 4.9 × 10^6^, *M*_w_ = 7.2 × 10^6^, PDI = 1.5 (containing 30% oligomeric segments, determined by the UV monitor); IR *ν* 3736, 3621, 3017, 3012, 2972, 2161, 2025, 1738, 1441, 1367, 1215, 1095, 1030, 828, 799, 671, 531 cm^−1^. The data of UV/vis, fluorescence/excitation spectra, and lifetime decays for the characterization are shown in Fig. 3.

#### Synthesis of PT2

The dibrominated TM (50 mg, 0.035 mmol), 5,5′-bis(4,4,5,5-tetramethyl-1,3,2-dioxaborolan-2-yl)-2,2′-bithiophene (14 mg, 0.034 mmol), K_3_PO_4_ (233 mg, 1.10 mmol), and Pd(PPh_3_)_4_ (20 mg, 50 mol%) were dissolved in dry DMF (10 mL) and the solution was stirred for 9 d at 90 °C under an N_2_ atmosphere. The solvent was removed under high vacuum. The residue was dissolved in DMF (1 mL), and the solution was slowly poured into *n*-hexane (20 mL). The precipitate obtained was filtered, collected, and dried under high vacuum to give PT2 (18 mg, 0.012 mmol in monomer unit) as an orange solid in 34% yield. ^1^H NMR (CDCl_3_, 400 MHz, 20 °C) *δ*_H_ 8.10–6.04 (br, 24H), 4.35–3.05 (br, 34H), 2.99–2.54 (br, 4H), 2.12–1.58 (br, 4H); ^13^C NMR (CDCl_3_, 400 MHz, 20 °C) *δ*_C_ 167.7, 146.0, 140.8, 140.6, 137.0, 131.1, 130.1, 129.8, 129.5, 129.2, 129.0, 126.2, 123.5, 120.3, 120.1, 72.9, 72.8, 72.6, 70.8, 70.7, 70.4, 70.2, 65.6, 63.8, 59.1, 29.8, 28.6, 27.5, 25.8, 25.3, 25.1, 24.6; *M*_n_ = 3.8 × 10^6^, *M*_w_ = 6.1 × 10^6^, PDI = 1.6 (containing 33% oligomeric segments, determined by the UV monitor); IR *ν* 3731, 3620, 2917, 2969, 2161, 1613, 1577, 1507, 1467, 1442, 1385, 1254, 1209, 1094, 1029, 1005, 829, 800, 686, 530 cm^−1^. The data of UV/vis, fluorescence/excitation spectra, and lifetime decays for the characterization are shown in Fig. 3.

## Results and discussion

### Design and synthesis of polythiophene chemosensors (PT1 and PT2)

**Fig. 2:**
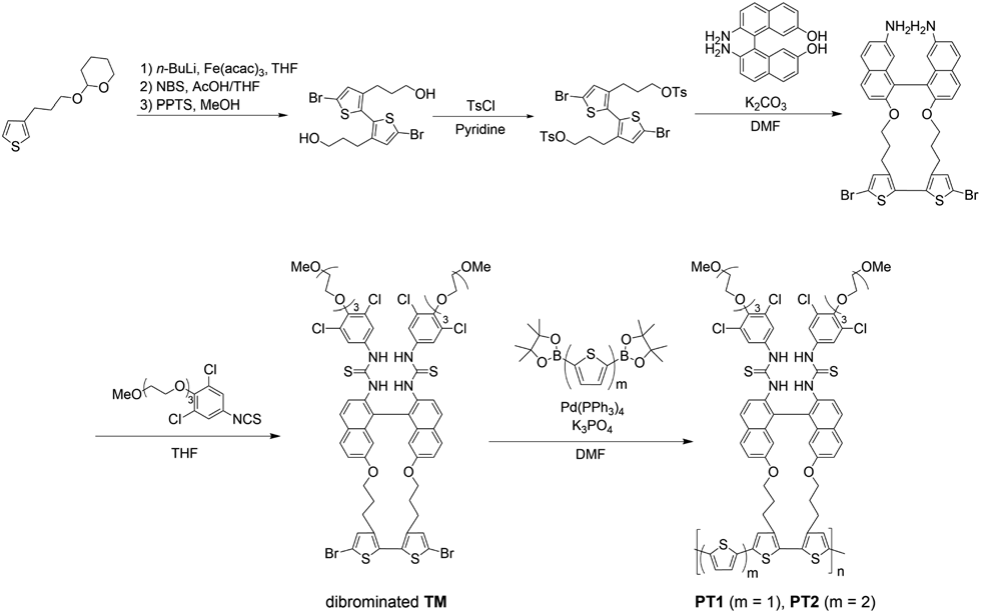
Synthesis of novel polythiophene chemosensors (PT1 and PT2).

In order to clearly estimate the scope and limitations of the PT-based SASS, we carefully designed monothiophene- and bithiophene-spaced PT1 and PT2, respectively, enabling us to understand the effects on the SASS mechanism that may be different from those observed in the direct TM-connected PT0. As shown in Fig. S9 in ESI,† decidedly, the Hartree–Fock calculations for the optimized structures of the model compounds for both the PTs also support the validity of the molecular design shown in this study. As can be seen in Table 1, the dihedral angles of models PT1 and PT2 are 73–78° in the recognition units, which are slightly larger than those (59–64°)^30^ of the model PT0 but twisting *syn* forms. This indicates that the bisthiourea moieties in both the PTs may function as the tweezer recognition site. The synthetic scheme is shown in Fig. 2. The THP-protected 3-(thiophen-3-yl)propan-1-ol was selected as a starting material since the propyl group is known as the most suitable alkyl chain length^30^ for better converting molecular recognition information upon the bisthiourea site in the repeating unit to dihedral angle changes at the bithiophene scaffold. This is also supported by the calculations (see the data in Table 1). Thus, the propylated thiophene was first dimerized with *n*-BuLi and Fe(acac)_3_, brominated at the α-position of the bithiophene and deprotected. The dibromobithiophene thus obtained was tosylated with tosyl chloride. The ditosylate was reacted with the binaphthyl transducer under a high dilution condition to afford the cyclic compound. The cyclic dibromobithiophene was further reacted with the isothiocyanate to give the dibrominated triad monomer (TM) possessing the bisthiourea moiety. The dibromo-monomer was then polymerized through the Suzuki–Miyaura coupling, rather than the conventional Stille coupling for the PT synthesis,^31^ with diboronic pinacol ester-monothiophene and -bithiophene to afford PT1 and PT2, respectively.

**Table 1:** Dihedral angles of the model PTs with the optimization by Gaussian 16 calculations^a^

Compd	Dihedral angle/°				
	Recognition units^b^		Spacer units^b^		
	*θ*_a_	*θ*_b_	*θ*_1_	*θ*_2_	*θ*_3_
PT1_5-mer_	73.58	78.28	173.63	176.42	—
PT1_5-mer_ + isophthalate^c^	79.51	67.19	117.97	157.16	—
PT2_6-mer_	73.85	78.58	175.22	171.98	173.22
PT2_6-mer_ + isophthalate^c^	82.11	71.60	133.91	135.95	153.74

a Basis set: HF/6-31G.

b See Fig. S9 in ESI for the optimized structures with the notations.

c Counter cation was omitted for simplicity during the calculation.

### Photophysical properties of PT1 and PT2

**Fig. 3:**
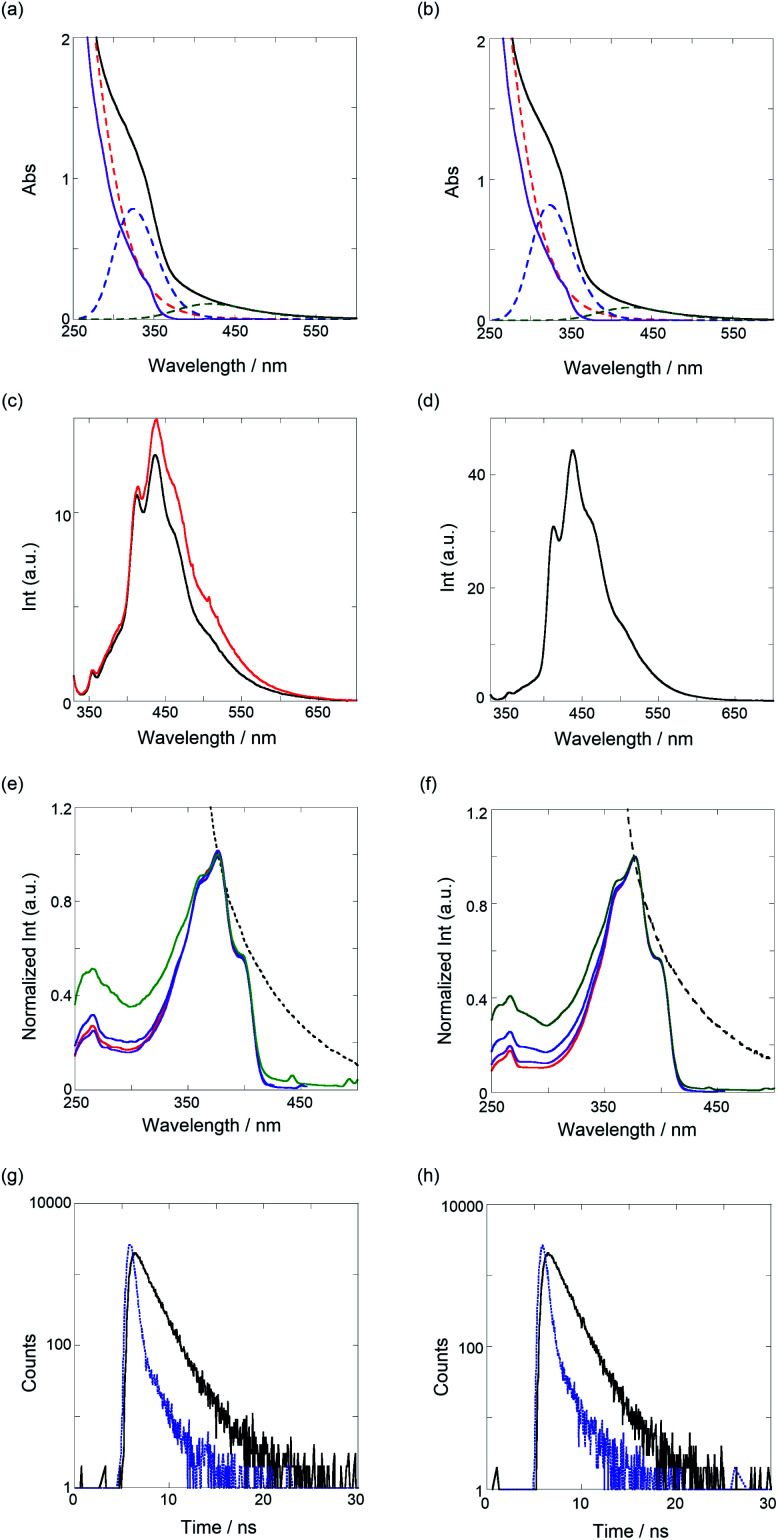
Top panels: UV/vis spectra of (a) PT1 (52.4 μM) and (b) PT2 (51.5 μM, black solid lines) and TM (normalized spectrum at 340 nm, purple solid lines) and results of waveform separation (colored dotted lines). The second panels from top: fluorescence spectra (*λ*_ex_ 320 nm) of (c) PT1 in the absence (5.23 μM, black) and presence of TBA (21 μM, red) and (d) PT2 (5.15 μM). The third panels from top: excitation spectra of (e) PT1 (normalized UV/vis spectrum, black dotted line) and (f) PT2 (normalized UV/vis spectrum, black dotted line), monitored at 412 (red), 436–438 (purple), 465–466 (blue), and 510 nm (green). Bottom panels: time-correlated fluorescence decays (*λ*_ex_ 340 nm, black solid lines) of (g) PT1 (5.23 μM, *λ*_em_ 436 nm) and (h) PT2 (5.15 μM, *λ*_em_ 438 nm) at room temperature, where the blue dotted lines show the instrument response function (IRF).

Next, we investigated the optical properties of PTs *via* UV/vis, fluorescence/excitation spectra, and fluorescence lifetime measurements in CHCl_3_. As shown in Fig. 3a and b, the UV/vis spectra of both the PTs broadened at around 600 nm, the edge of which was assignable to the π–π* transition of PT. Certainly, the spectra can be applicably separated to a sum of three species as a Gaussian waveform. The red dotted lines observed at the shorter wavelength almost overlap with the spectra of TM (purple solid line), in which the binaphthyl and bithiophene chromophores are contained. Thus, the blue dotted lines peaked at 324 nm, which can be ascribed to an oligomeric segment in the polymer, and the green ones that peaked at 420 nm for PT1 and 424 nm for PT2 are the π–π* transitions. According to the equation for the effective conjugation length (*n*_e_) of PT (*E* (eV) = 2.49 + 3.14/*n*_e_),^36^*n*_e_ values of the oligomer, PT1, and PT2 were determined as 2.3, 6.8, and 7.2, respectively, calculated from the extrema of the blue and green dotted lines. These *n*_e_ values, except for the oligomer, are quite larger and more extended than the *n*_e_ (2.8) of PT0 due to the mono- and bithiophene spacers, which can adjust as energetically stable dihedral angles (more *anti*) between TM and spacers. As shown in Table 1, definitely, the dihedral angles at the spacer units are 171–176°, almost *anti* forms.

As shown in Fig. 3c and d (black lines), the band-broadened fluorescence spectra at 400–650 nm were observed. The broad emission bands are based on fluorescent species of oligomeric chromophore segments through an energy transfer in the conjugated polymer chain.^37^ The excitation spectra (see Fig. 3e and f) were observed in the range of 300–420 nm, the main emissive species of which comes from a 5-mer (the extremum at 398 nm) based on the *n*_e_ equation. The UV/vis and fluorescence maxima are summarized in Table 2. To further elucidate the excited-state behavior of PTs, we measured fluorescence lifetimes by the time-correlated single-photon-counting method, shown in Fig. 3g and h. The fluorescence decay profiles were reasonably fitted to a sum of two and one exponential functions to give 1.4 and 0.3 ns for PT1 and 1.3 ns for PT2, respectively, as listed in Table 2. Based on the results of the excitation spectra, the longer lived excited species (1.3–1.4 ns) is ascribable to the main emissive oligomer (*ca.* 5-mer) and the shorter one to another oligomeric fragment (<5-mer).

**Table 2:** Photophysical properties of PTs^a^

Compd	UV/vis *λ*_max_/nm	FL *λ*_em_/nm	*n* b ^,^ c	*τ*_1_ b/ns	*A*_1_ b	*τ*_2_ b/ns	*A*_2_ b	*χ* ^2^ b
PT1	420	436	2	1.4	0.90	0.3	0.10	1.1
PT2	424	437	1	1.3	1.00			1.1

a Measured in CHCl_3_ at 25 °C, unless noted otherwise.

b Fluorescence lifetime (*τ*_i_/ns) and relative abundance (*A*_i_) of each component determined by the time-correlated single-photon-counting method in nondegassed CHCl_3_ solution at room temperature (*λ*_ex_ 340 nm, *λ*_em_ 436–438 nm); a lifetime of the TM was omitted due to over the measurement limitation (∼ps) of the fluorescence lifetime apparatus employed in this study.

c Number of components.

An interesting fluorescence enhancement was also observed upon the addition of TBA as a guest molecule, as shown in Fig. 3c (red line). In the absence of the guest molecule as an initial stage, the fluorescence was suppressed by a photoinduced electron transfer (PET) from the amines in the thiourea to the fluorescent PT chromophore. Then, the addition of a small amount of TBA (11% complexation determined by the binding constant) recovered the fluorescence intensity due to the inhibition of the intra-PET process, as was the case with PT0.^30^ This result implies that these PTs may be useful as a turn-on fluorescence chemosensor.

### Mechanistic origins of SASS in PT1 and PT2

**Fig. 4:**
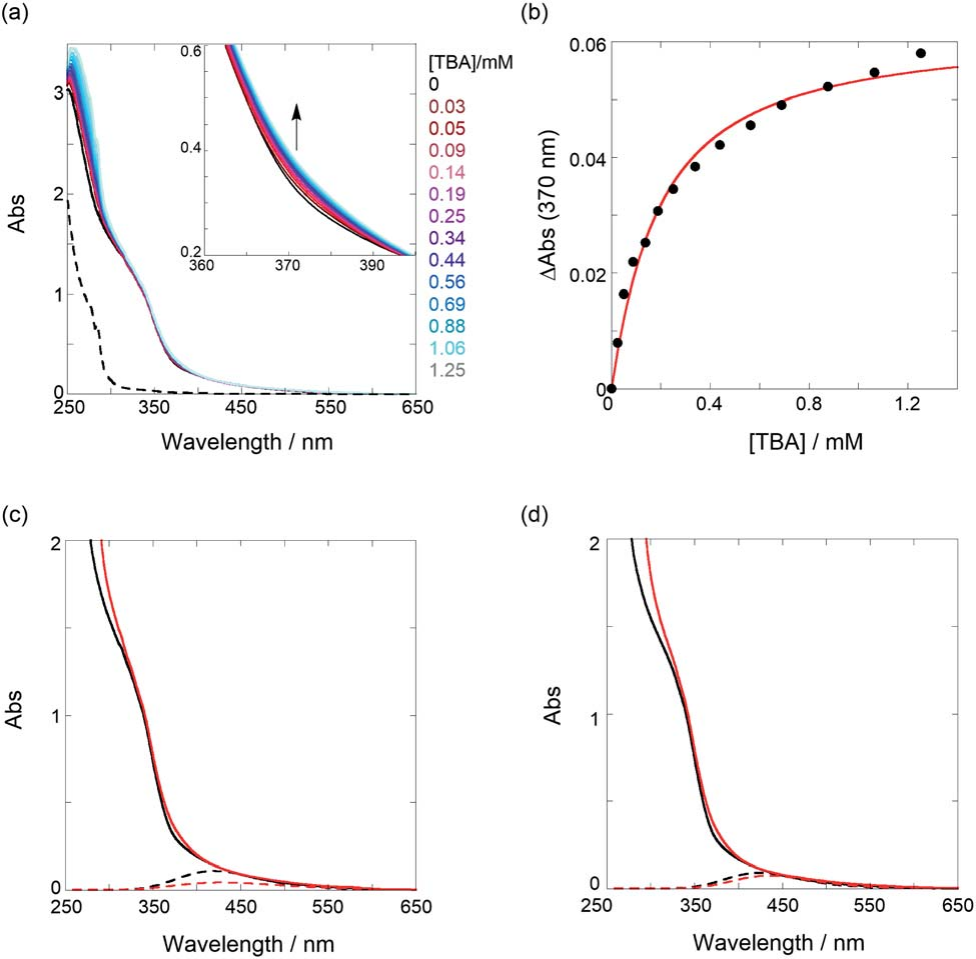
(a) UV/vis spectral changes of a chloroform solution of PT1 (52.4 μM, black) upon gradual addition of TBA (0.03–1.25 mM, colored lines); the dotted line represents the UV spectrum of TBA only. (b) Nonlinear least-squares fitting, assuming a 1 : 1 model, to determine the binding constant. UV/vis spectra of (c) PT1 (52.4 μM) and (d) PT2 (51.5 μM) in the absence (black solid lines) and presence (red solid lines) of TBA (1.25 mM for PT1 (88% complexation) and 2.18 mM for PT2 (93% complexation)) and results of waveform separation (dotted lines); the spectra without TBA (black solid and dotted lines) in (c and d) are identical to those shown in Fig. 3a and b. Full results of waveform separation for the spectra with TBA are given in Fig. S13 in ESI.†

Last, we examined the binding behavior of both the PTs using TBA and TBP salts as model analytes and then attempted to explain the mechanistic origins of the SASS operated in PT. As shown in Fig. 4a, the gradual addition of TBA to a CHCl_3_ solution of PT1 at 25 °C caused slight band-broadening, hyperchromic effect, and bathochromic shift, indicating a supramolecular complexation of TBA *via* a perturbation of the chromophores in PT1. The TM unit can trap isophthalates through the 1 : 1 complexation stoichiometry with binding constants (*K*) determined by the Job plot.^30^ Thus, in Fig. 4b, a nonlinear least-squares fitting, assuming the 1 : 1 complexation, was subjected to the UV/vis titration to give *K* for TBA as 6030 M^−1^ (Δ*G*° as −21.6 kJ mol^−1^). Also, the same analysis of the UV/vis titration of the PTs gave the *K* values for TBA and TBP listed in Table 3 (Fig. S10–S12 in ESI†). Interestingly, the *K* values dropped sharply by a factor of 0.035–0.042, in sharp contrast to the amplification (5–15 times) of PT0. This is a rather contrasting behavior despite using the same repeating TM unit.

**Table 3:** Binding constants (*K*) and free energy changes (Δ*G*°) for 1 : 1 complexation of isophthalates with PTs^a^

Chemosensor	Analyte	*K*/M^−1^	Δ*G*°^b^/kJ mol^−1^
TM	TBA	159 000 ± 11 300	−29.5
	TBP	198 000 ± 39 600	−30.2
PT1	TBA	6030 ± 690	−21.6
	TBP	8310 ± 1480	−22.4
PT2	TBA	5790 ± 570	−21.5
	TBP	6840 ± 1130	−21.9

a The binding constants of PTs were determined using monomer unit concentration.

b Gibbs energy changes at 298 K are listed.

To elucidate the origins of the contrasting binding amplification *vs.* the inhibition, the UV/vis spectral changes in the absence and presence of TBA were examined. As shown in Fig. 4c and d, the spectra, upon the addition of an adequate amount of TBA (≥88% complexation), showed appreciable bathochromic shifts, indicating an extension of *n*_e_. The waveform separations by the addition of TBA strictly afforded the extrema changes from 420 nm to 429 nm for PT1 and from 424 nm to 435 nm for PT2, respectively. According to the equation, the *n*_e_ value of 6.8 (7.2) was changed to 7.8 (8.7) for PT1 (for PT2). These results can be deduced from facts based on the changes in the dihedral angle of PT backbones. For instance, from the data of PT1 listed in Table 1, the angle in the tweezer increased from 73° to 79° upon the addition of TBA, and then, the other binding site slightly closed (from 78° to 67°). Thus, the averaged angle changes in the recognition unit are almost negligible (76° → 73°) before and after the interaction of TBA, for which a relaxation of the thiophene spacer unit is highly likely to be responsible. Namely, freely rotating/changing the dihedral angles between the spacers (*θ*_1_ and *θ*_2_) may inhibit a tighter tweezer mode at the neighbor recognition position by the first binding. Also, the averaged angle of 73° after the interaction of TBA is demonstrably larger than that of 31° in the PT0 for the tighter tweezer (the amplification mode), eventually causing the drastic decrease of the binding constant. This trend can also be seen in the case of PT2. The averaged angle changes at the recognition unit are almost the same as 76° before and after the addition of TBA, which is consistent with the case of PT1. These results suggest that the thiophene spacers between the recognition sites play a critical role in amplification sensing. Therefore, it can be emphasized that the binding behavior of target analytes can be drastically controlled, *i.e.*, amplification or inhibition, just by changing the conformationally flexible spacers.

## Conclusion

We have developed the newly synthesized polythiophene chemosensors (PT1 and PT2) that afford information on chemical sensing based on the conformational changes of polymer backbones upon inclusion of salt guests. The binding constants obtained in this study were rather inhibited by a factor of 0.035–0.042, and thus, very different from those observed in the previous amplification chemosensor (5–15 times). The relaxation of the recognition information through the thiophene spacer units is a critical factor that governs allosteric chemical sensing. The present study provided new guidelines for an effective molecular design of SASS-operative chemosensors, and hence, can be expanded to other polymer chemosensors that show an allosteric amplification.

## Conflicts of interest

The authors declare no competing financial interest.

## Supplementary Material

RA-011-D1RA05795B-s001
