# Supplementary material for: The factors that govern the allosteric chemical sensing of polythiophene chemosensors: scope and limitation toward signal-amplification sensing
Source: RSC Adv. 2021 Sep 14;11(48):30472–8. doi: 10.1039/d1ra05795b (PMC9041329; doi:10.1039/d1ra05795b)
Supplement: RA-011-D1RA05795B-s001 [file RA-011-D1RA05795B-s001.pdf]

Electronic Supplementary Material (ESI) for RSC Advances.
This journal is © The Royal Society of Chemistry 2021

Electronic Supplementary Information

The factors that govern the allosteric chemical sensing of polythiophene chemosensors: scope and limitation toward signal-amplification sensing

Tomoaki Tsuchiya,[a] Hiroaki Mizuno[a] and Gaku Fukuhara[*ab]*

[a]Department of Chemistry, Tokyo Institute of Technology, 2-12-1 Ookayama, Meguro-ku, Tokyo 152-8551, Japan

[b]JST, PRESTO, 4-1-8 Honcho, Kawaguchi, Saitama 332-0012, Japan

*E-mail: gaku@chem.titech.ac.jp

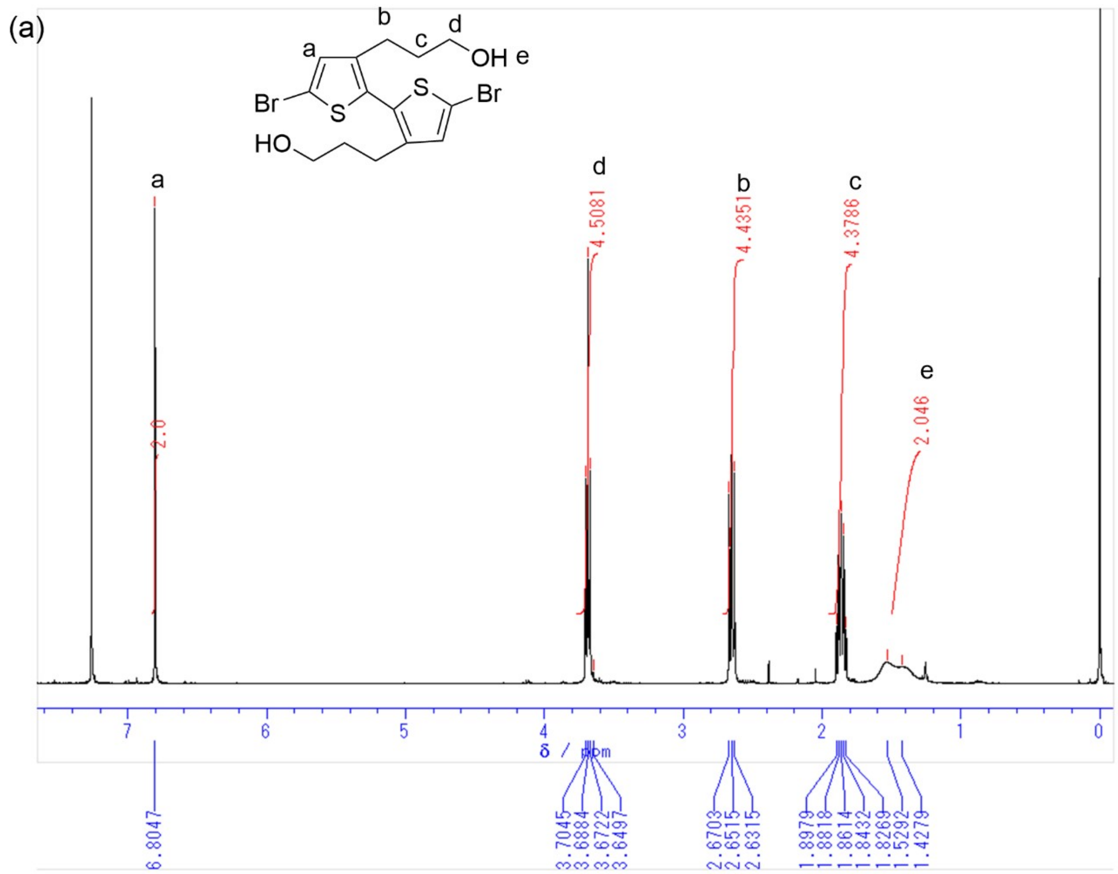

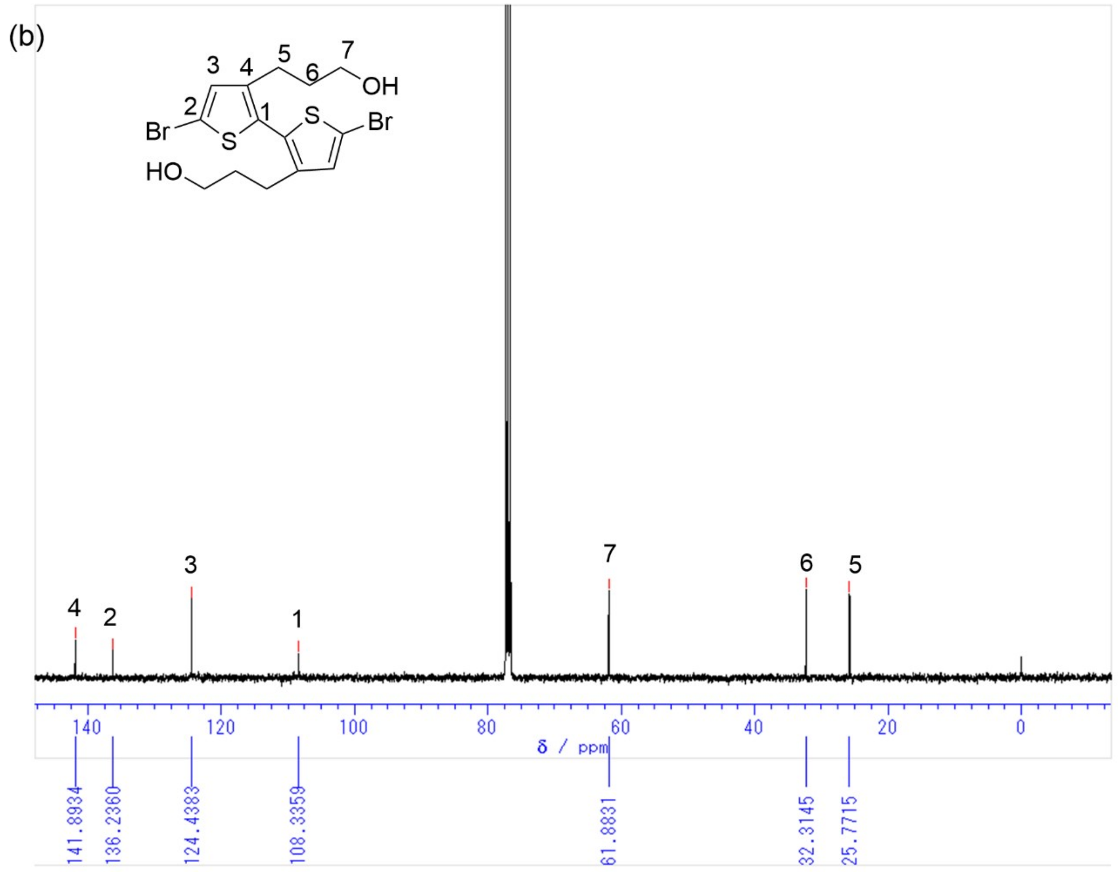

Figure S1. (a) ^{1}H (400 MHz) and (b) ^{13}C NMR spectra (100 MHz) of 3,3'-(5,5'-dibromo-[2,2'-bithiophene]-3,3'-diyl)bis(propan-1-ol) in $CDCl_3$.

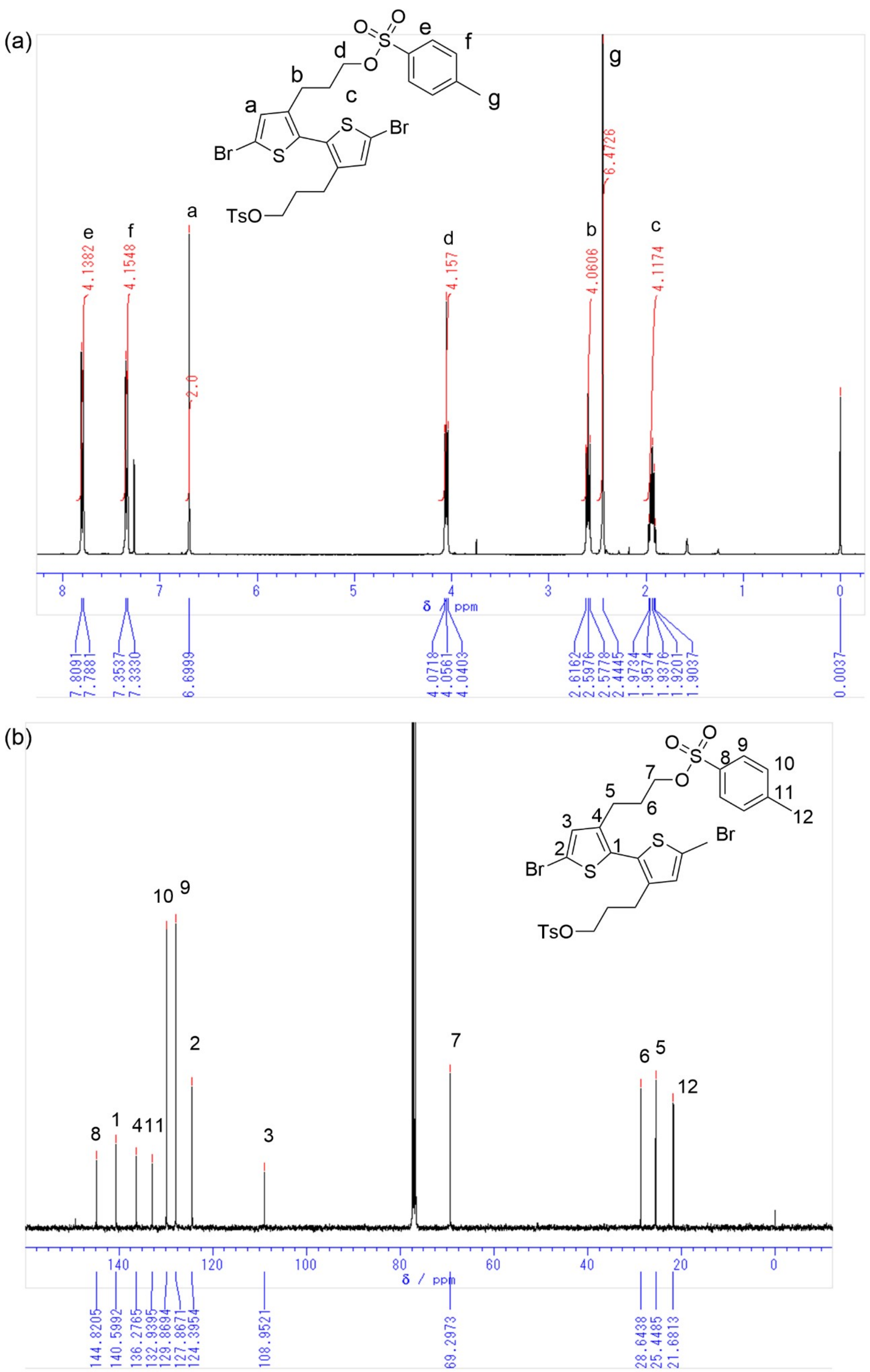

Figure S2. (a) ^{1}H (400 MHz) and (b) ^{13}C NMR spectra (100 MHz) of (5,5'-dibromo-[2,2'-bithiophene]-3,3'-diyl)bis(propane-3,1-diyl) bis(4-methylbenzenesulfonate) in $CDCl_3$.

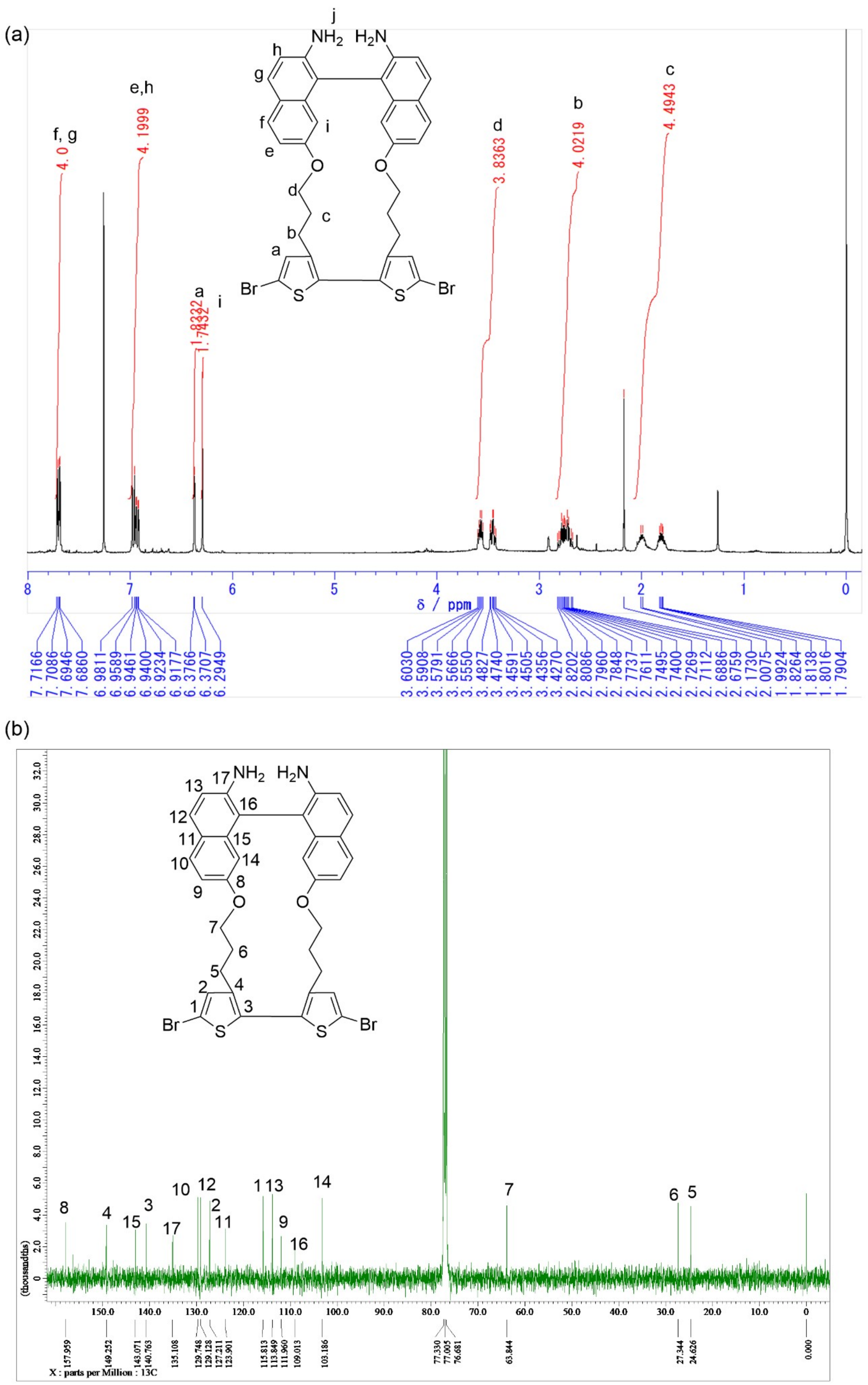

Figure S3. (a) ^{1}H (400 MHz) and (b) ^{13}C NMR spectra (100 MHz) of binaphthyl-dibromobithiophene conjugate in $CDCl_3$.

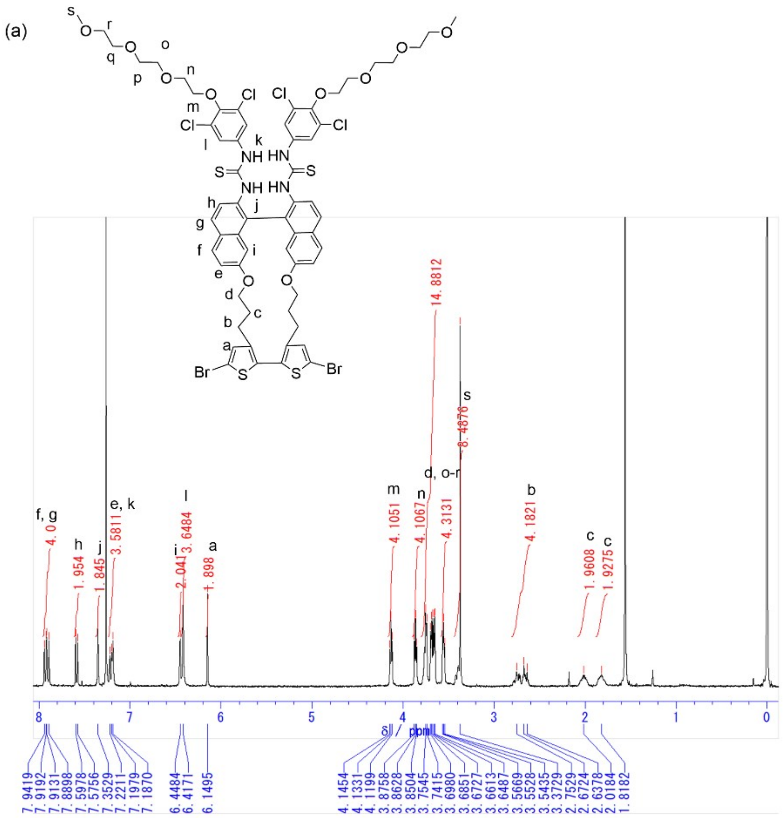

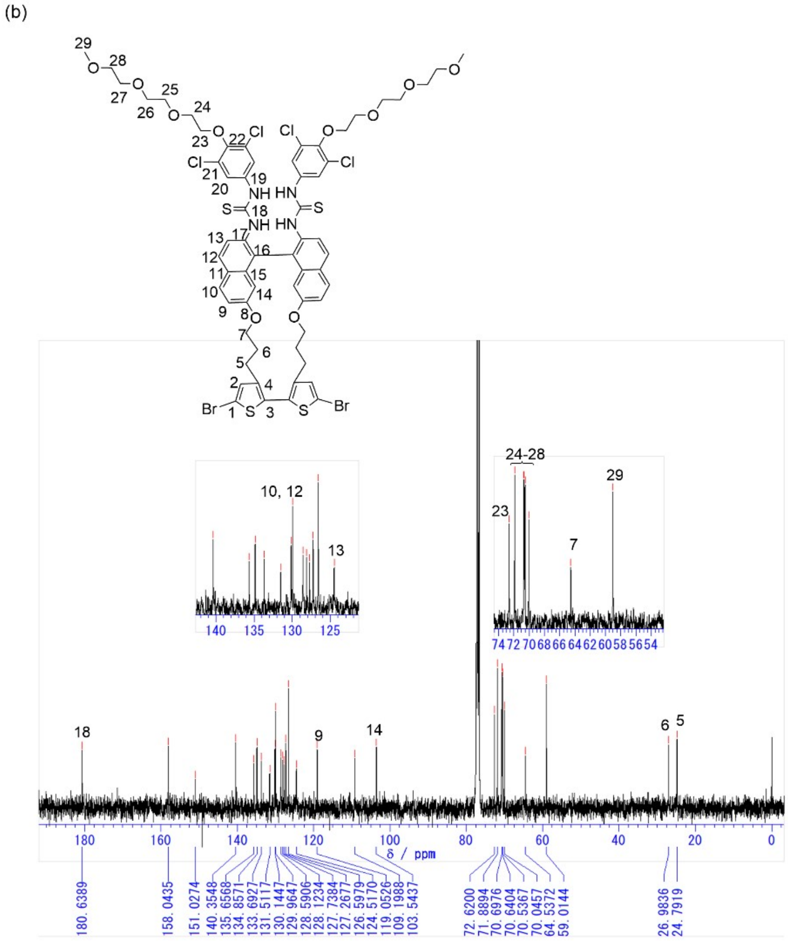

Figure S4. (a) ^{1}H (400 MHz) and (b) ^{13}C NMR spectra (100 MHz) of bisthiourea-binaphthyl-dibromobithiophene conjugate (dibrominated **TM**) in $CDCl_3$.

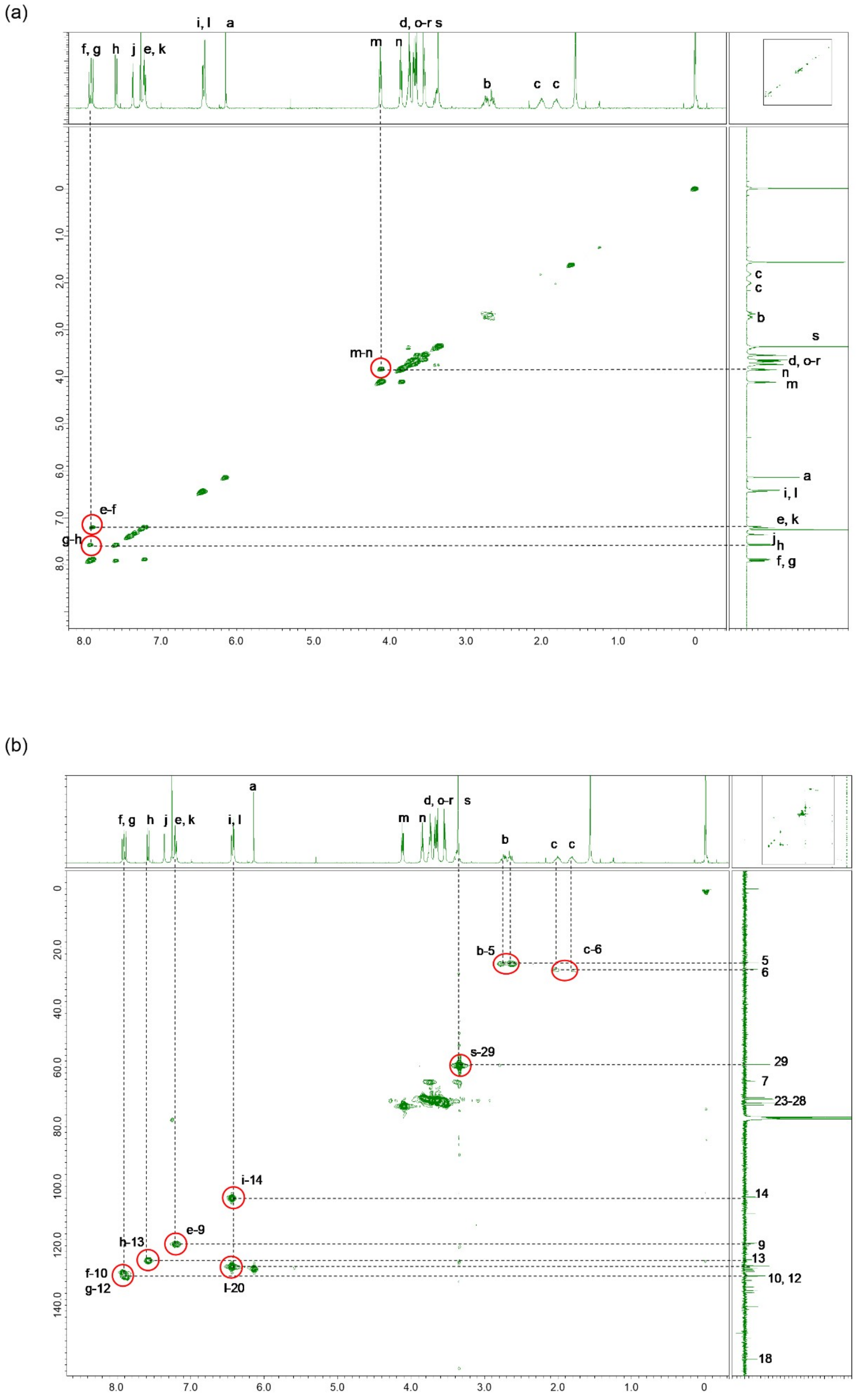

Figure S5. (a) COSY and (b) HMQC spectra of bisthiourea-binaphthyl-dibromobithiophene conjugate (dibrominated **TM**) in $CDCl_3$.

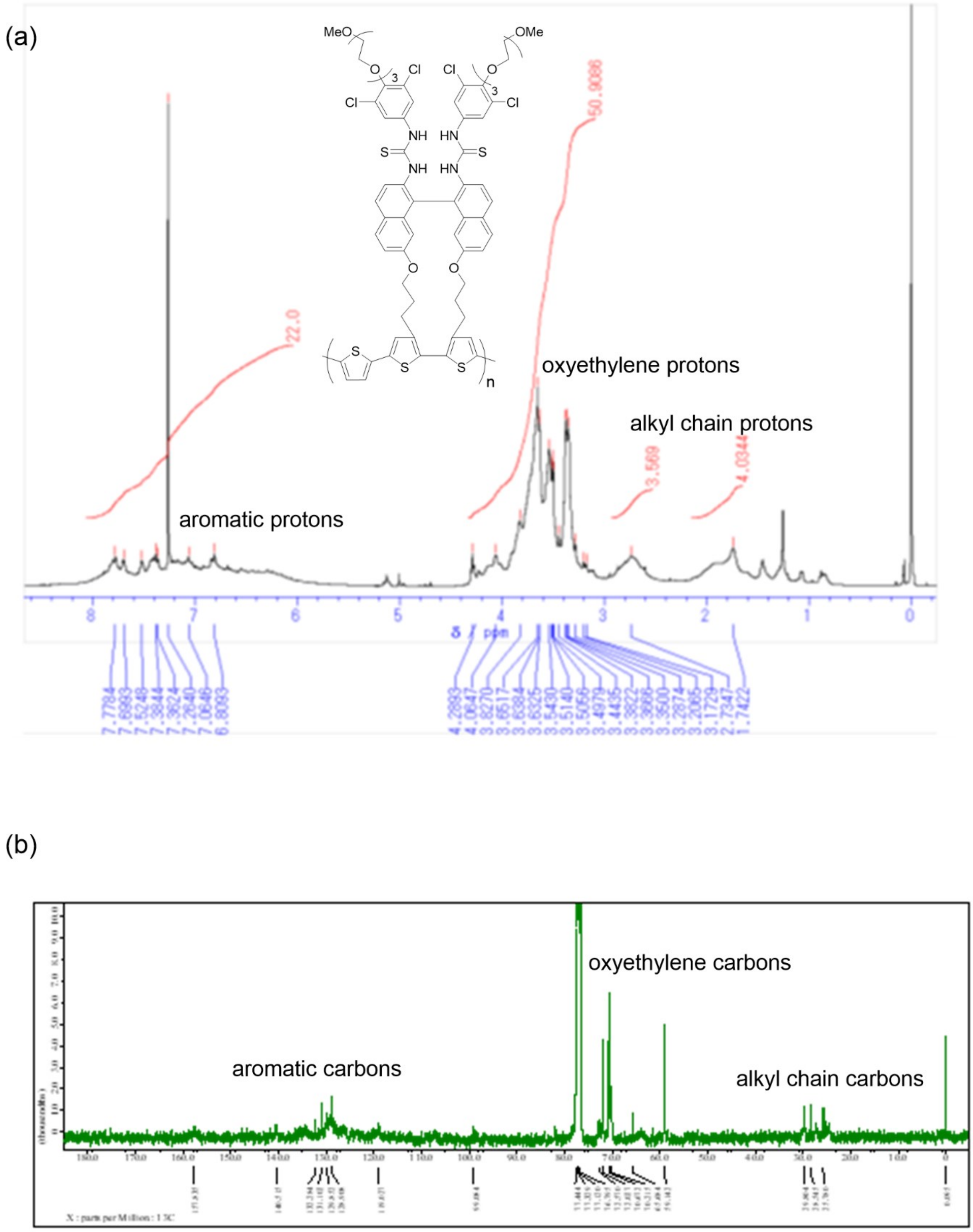

Figure S6. (a) ^{1}H (400 MHz) and (b) ^{13}C NMR spectra (100 MHz) of **PT1** in $CDCl_3$.

(a)

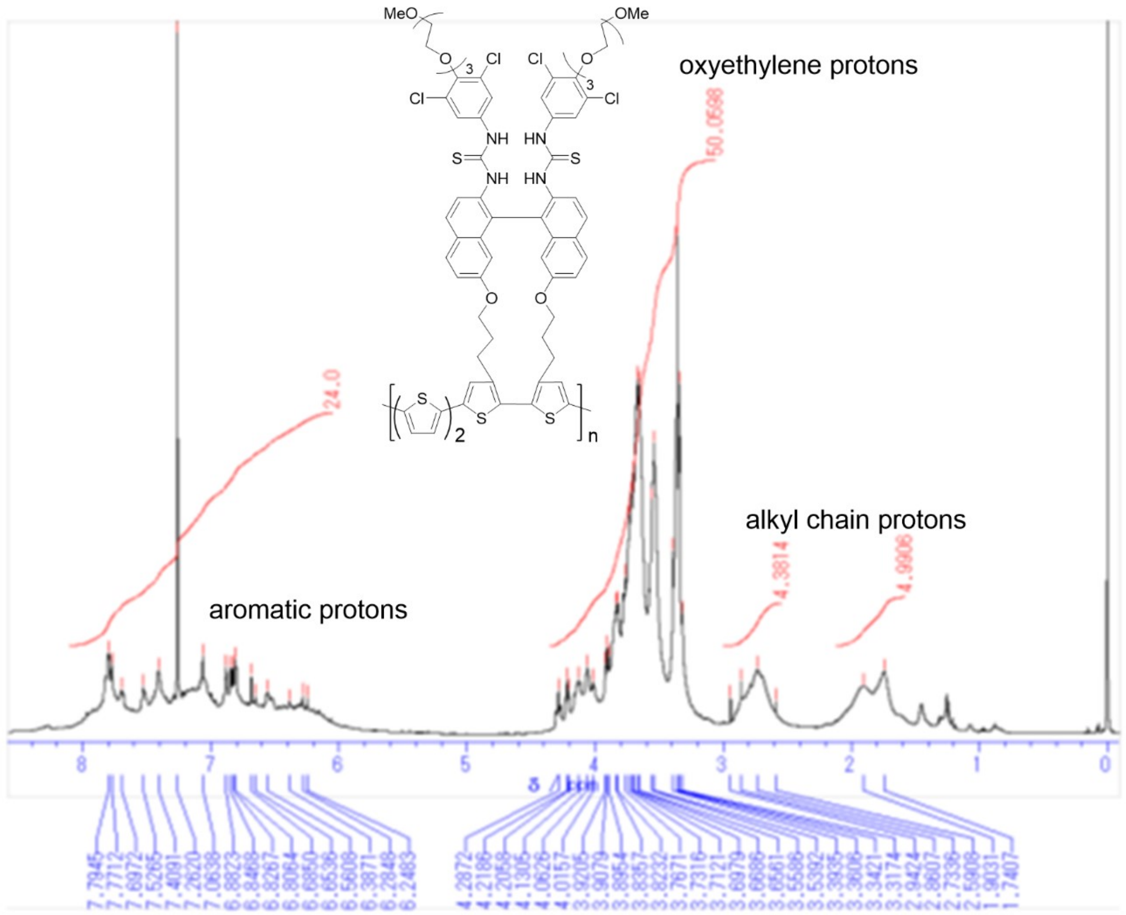

(b)

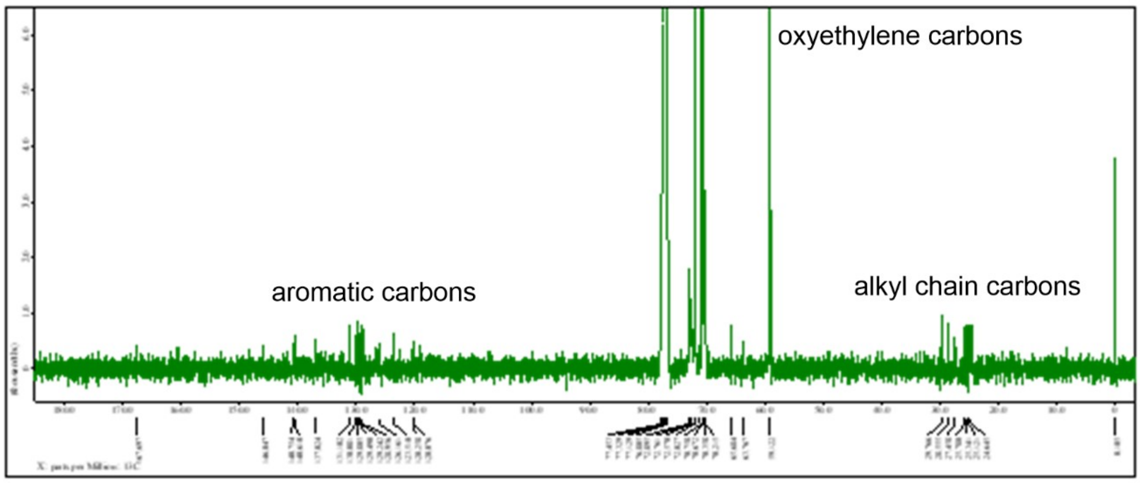

Figure S7. (a) ^{1}H (400 MHz) and (b) ^{13}C NMR spectra (100 MHz) of **PT2** in $CDCl_3$.

(a)

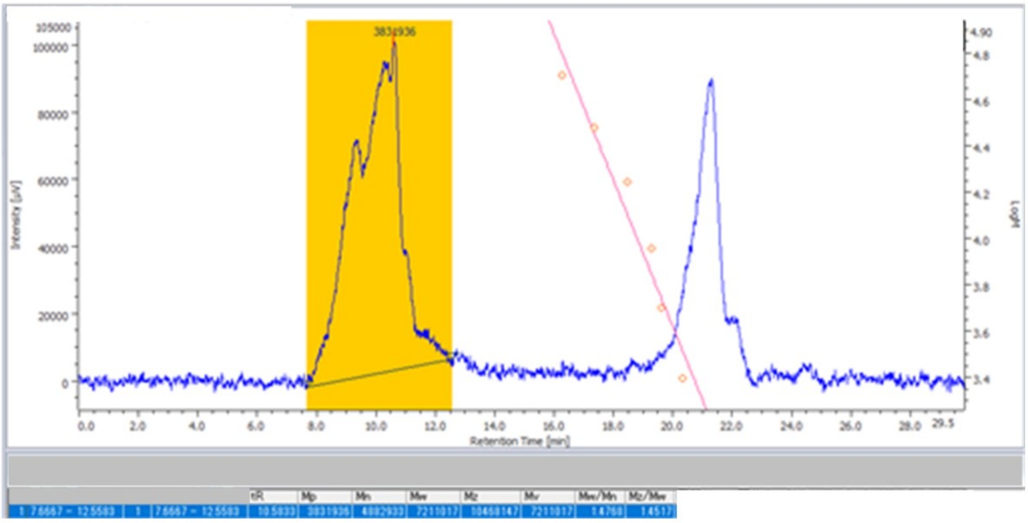

(b)

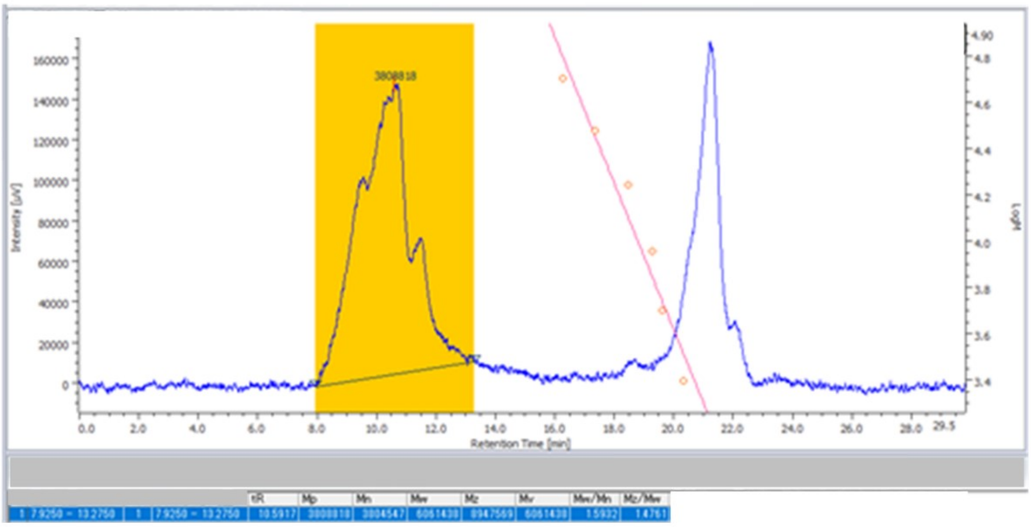

Figure S8. Analytical GPC chromatograms of (a) **PT1** and (b) **PT2**.

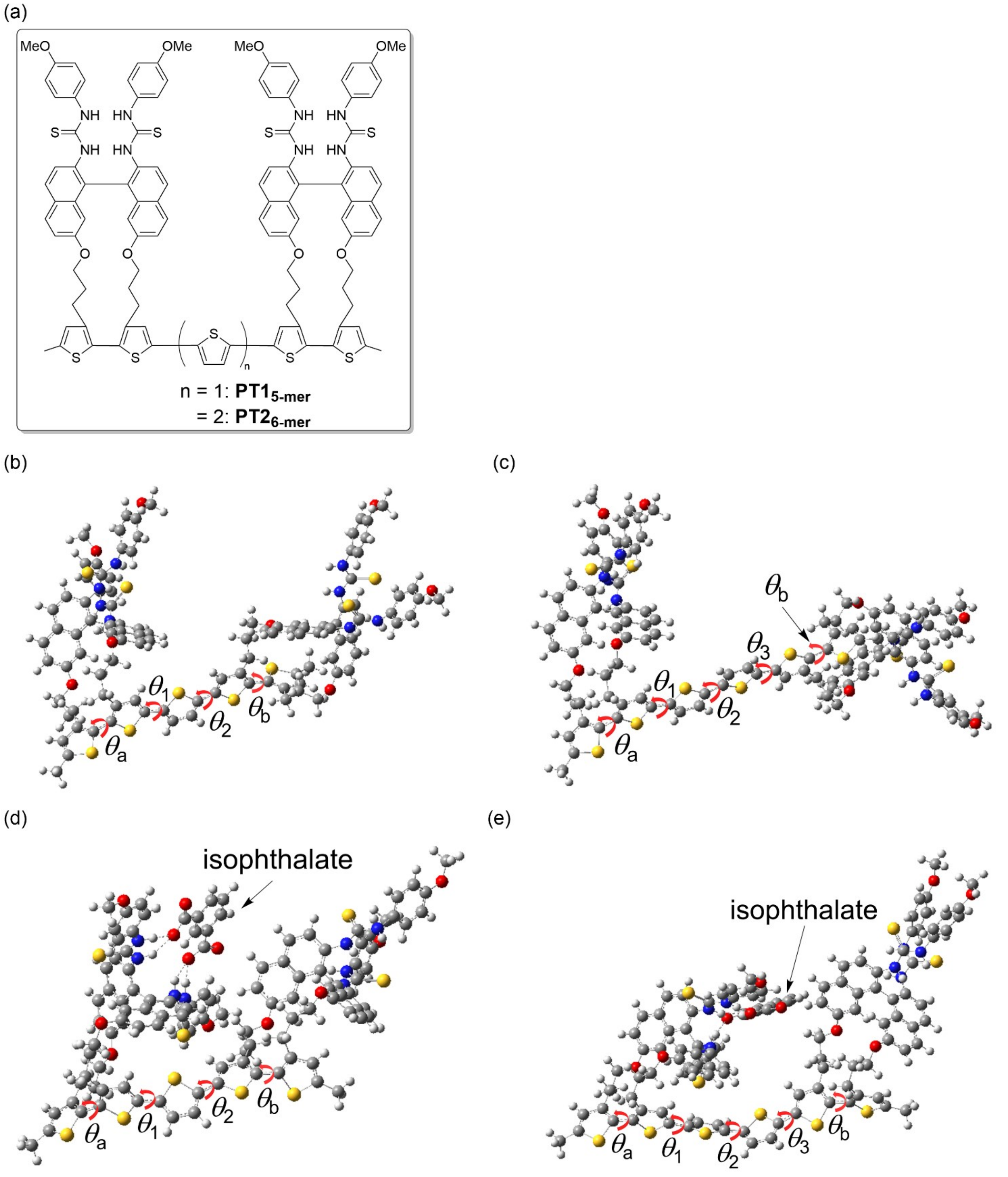

Figure S9. (a) Chemical structures of model compounds for **PT1** as **PT1$_{5\text{-mer}}$** and for **PT2** as **PT2$_{6\text{-mer}}$**, where the oxyethylenes and the chloride groups were omitted and both ends at α position of thiophenes were methylated for the simplification reason. The optimized structures of **PT1$_{5\text{-mer}}$** and **PT2$_{6\text{-mer}}$** via Gaussian 16 calculation (HF/6-31G) in the absence (b,c) and presence of isophthalate (d,e); counter cation was omitted for clarification.

Summary of Structure Optimization

PT1$_{5\text{-mer}}$

Symbol	X	Y	Z
C	0.911679	-4.12421	1.787766
C	0.064366	-3.32224	1.112746
C	-1.14875	-2.97063	1.812063
C	-1.23149	-3.51815	3.043415
S	0.236923	-4.51909	3.409957
C	-2.26856	-3.38353	4.060864
C	-3.44948	-4.02489	4.186488
C	-4.18607	-3.62548	5.37022
C	-3.58976	-2.70235	6.139732
S	-2.0055	-2.22938	5.439692
C	-4.74574	-0.7506	-1.37659
C	-5.71659	-1.14198	-2.25008
C	-7.07961	-1.16985	-1.87884
C	-7.44059	-0.78081	-0.56939
C	-6.41812	-0.36619	0.319428
C	-5.10822	-0.35521	-0.07005
C	-8.08839	-1.60054	-2.77667
C	-9.3921	-1.61718	-2.4087
C	-9.77036	-1.18971	-1.10894
C	-8.82659	-0.81126	-0.18528
C	-8.26318	-3.66905	2.96491
C	-8.45301	-2.75937	1.97037
C	-8.98329	-1.47263	2.256962
C	-9.29954	-1.15858	3.597129
C	-9.10804	-2.1393	4.602753
C	-8.60859	-3.36884	4.301258
C	-9.20337	-0.48955	1.231731
C	-9.72218	0.733441	1.57865
C	-9.98936	1.057609	2.932694
C	-9.7922	0.133927	3.905387
C	-2.17923	-2.06249	1.188375
O	-4.197	0.021569	0.888875
O	-7.76118	-4.91738	2.642465
N	-11.1322	-1.24096	-0.71591

N	-9.91387	1.711752	0.568558
C	-12.233	-0.84425	-1.39959
C	-10.9954	2.499915	0.357258
N	-13.3807	-1.05515	-0.73669
S	-12.1967	-0.10393	-2.9796
N	-10.8602	3.331955	-0.68652
S	-12.4462	2.441075	1.324124
C	-14.697	-0.71778	-1.19033
C	-11.8467	4.249108	-1.17512
C	-15.428	-1.63182	-1.9231
C	-16.723	-1.33503	-2.32986
C	-17.2773	-0.11026	-1.99145
C	-16.5441	0.811225	-1.25158
C	-15.2573	0.509951	-0.85379
C	-11.8527	5.563566	-0.72303
C	-12.7688	6.465126	-1.22415
C	-13.6897	6.053525	-2.18205
C	-13.689	4.743629	-2.63535
C	-12.7617	3.842057	-2.12687
C	-2.81348	0.348087	0.654818
C	-6.51022	-5.35495	3.219032
C	-1.92325	-0.57259	1.476308
C	-5.31451	-4.68819	2.547512
C	-3.96581	-5.06041	3.2158
H	0.266547	-2.9727	0.119786
H	-5.14103	-4.03765	5.624042
H	-3.72328	-0.75818	-1.68943
H	-5.44672	-1.4454	-3.24399
H	-7.80613	-1.92537	-3.7599
H	-10.1474	-1.94298	-3.08732
H	-9.37452	-1.89804	5.614321
H	-8.49095	-4.11678	5.060011
H	-10.359	2.030405	3.168523
H	-10.0033	0.380443	4.928285
H	-3.16611	-2.31597	1.546574
H	-2.17999	-2.23228	0.115137
H	-11.2689	-1.50462	0.235108
H	-9.2065	1.719338	-0.13287

H	-13.3267	-1.41196	0.192099
H	-10.0206	3.280443	-1.22064
H	-14.9863	-2.57205	-2.18835
H	-17.2718	-2.0543	-2.90179
H	-16.9973	1.749274	-1.00505
H	-14.6805	1.219063	-0.29371
H	-11.148	5.868838	0.025155
H	-12.7961	7.481561	-0.88917
H	-14.3932	4.408734	-3.36852
H	-12.7602	2.824412	-2.4644
H	-2.68691	1.376155	0.962308
H	-2.57329	0.281135	-0.39618
H	-6.49686	-6.42192	3.061181
H	-6.52103	-5.17059	4.284966
H	-2.08566	-0.38223	2.529709
H	-0.88849	-0.31926	1.261063
H	-5.44553	-3.61275	2.554432
H	-5.30477	-4.99264	1.507478
H	-4.06638	-6.01115	3.735827
H	-3.21942	-5.21767	2.450125
C	-15.5887	6.717213	-3.57105
H	-16.1257	7.638918	-3.71333
H	-15.1672	6.392058	-4.51321
H	-16.2597	5.958661	-3.18996
C	-19.3992	-0.56395	-3.11876
H	-20.3118	-0.00851	-3.24821
H	-18.962	-0.7771	-4.08558
H	-19.6081	-1.49065	-2.60001
O	-14.5609	7.015638	-2.62421
O	-18.5452	0.277887	-2.34326
H	-8.18966	-3.03226	0.970691
H	-6.64792	-0.06446	1.319149
C	10.42645	-8.24639	-3.58927
C	10.95319	-7.07956	-3.18872
C	10.0124	-6.1247	-2.63506
C	8.741955	-6.5773	-2.6287
S	8.651381	-8.26046	-3.31079
C	7.515887	-5.93337	-2.17174

C	6.774711	-4.9666	-2.75619
C	5.589974	-4.62973	-2.00029
C	5.41446	-5.32078	-0.85735
S	6.762717	-6.48917	-0.61838
C	12.62295	-0.70458	-1.28922
C	12.70583	0.602355	-1.67222
C	11.61355	1.486231	-1.52926
C	10.4228	1.009585	-0.93382
C	10.35205	-0.35273	-0.55237
C	11.41243	-1.19219	-0.75185
C	11.68242	2.834711	-1.96784
C	10.62233	3.665517	-1.82638
C	9.427205	3.199558	-1.21824
C	9.318953	1.912531	-0.7619
C	6.08298	-0.20068	-2.71411
C	7.102556	0.463777	-2.10384
C	7.010725	0.813168	-0.73067
C	5.84361	0.461419	-0.01467
C	4.783201	-0.18328	-0.69882
C	4.893216	-0.50513	-2.01584
C	8.081625	1.469819	-0.0386
C	7.958406	1.693049	1.307186
C	6.794251	1.328429	2.022927
C	5.758743	0.743088	1.372584
C	10.46721	-4.773	-2.1464
O	11.22039	-2.51211	-0.41643
O	6.215687	-0.56615	-4.04221
N	8.370078	4.115298	-0.97911
N	9.059057	2.246323	2.036488
C	7.714811	4.879401	-1.87833
C	9.106425	3.443461	2.647119
N	6.784978	5.672725	-1.3123
S	8.02463	4.836517	-3.59048
N	10.24198	3.678417	3.325172
S	7.804932	4.609365	2.556084
C	5.908504	6.578138	-1.98328
C	10.58343	4.881821	4.022491
C	4.85671	6.113087	-2.75007

C	3.981555	7.002757	-3.35881
C	4.159818	8.366621	-3.18361
C	5.210468	8.839736	-2.40739
C	6.082893	7.947136	-1.81696
C	10.27997	5.01452	5.372823
C	10.65157	6.151787	6.058558
C	11.32883	7.170485	5.395548
C	11.63289	7.045517	4.048722
C	11.25578	5.894875	3.367615
C	12.16596	-3.55409	-0.71095
C	6.107999	-1.96202	-4.39674
C	11.3981	-4.84651	-0.92144
C	7.32792	-2.76272	-3.94708
C	7.118499	-4.29503	-4.0655
H	12.00045	-6.86524	-3.27673
H	4.895993	-3.88274	-2.32395
H	13.47196	-1.34554	-1.4034
H	13.61837	0.974533	-2.09833
H	12.58911	3.18851	-2.4205
H	10.66062	4.676644	-2.17002
H	3.888839	-0.42396	-0.157
H	4.082208	-0.979	-2.53194
H	6.74612	1.537497	3.071463
H	4.868238	0.470456	1.905457
H	9.616941	-4.15363	-1.90378
H	10.99366	-4.27802	-2.96029
H	8.064236	4.171718	-0.02945
H	9.884237	1.686981	2.039116
H	6.698002	5.642924	-0.31863
H	10.92289	2.951454	3.357812
H	4.729799	5.058474	-2.88744
H	3.180484	6.621228	-3.95752
H	5.32757	9.897516	-2.29074
H	6.905739	8.306553	-1.23038
H	9.744891	4.2324	5.874
H	10.42505	6.278804	7.096986
H	12.14888	7.821596	3.522543
H	11.4777	5.794586	2.32338

H	12.86182	-3.63559	0.114644
H	12.71987	-3.31277	-1.60902
H	6.015551	-1.963	-5.47116
H	5.195834	-2.37049	-3.98186
H	10.82056	-5.07762	-0.03479
H	12.1243	-5.64451	-1.04651
H	7.569921	-2.50599	-2.92321
H	8.175231	-2.45633	-4.5489
H	6.329534	-4.50279	-4.78551
H	8.012609	-4.75302	-4.46306
C	12.30108	9.406145	5.593103
H	12.40785	10.10353	6.405358
H	13.27707	9.151481	5.201102
H	11.69464	9.845196	4.811957
C	2.265184	8.975758	-4.60376
H	1.82576	9.910478	-4.90625
H	2.622359	8.441773	-5.47468
H	1.526278	8.377655	-4.08616
O	11.65916	8.25951	6.156404
O	3.344908	9.323067	-3.73617
H	7.973864	0.711257	-2.67274
H	9.453445	-0.76122	-0.13912
C	11.11649	-9.42488	-4.19469
H	10.99056	-10.3126	-3.58546
H	10.72959	-9.64626	-5.18279
H	12.17733	-9.22745	-4.28581
C	-4.08051	-2.08826	7.409881
H	-4.19445	-1.01481	7.311368
H	-5.04414	-2.50486	7.675224
H	-3.3961	-2.2736	8.22972
C	2.189346	-4.65337	1.368672
C	3.008204	-5.5123	2.010203
S	2.904019	-4.17505	-0.222
C	4.212773	-5.83193	1.302447
H	2.778127	-5.92483	2.971104
C	4.3407	-5.22388	0.105056
H	4.952464	-6.49854	1.695864

Total energy = -8677.403739 Hartree

$PT2_{6\text{-}mer}$

Symbol	X	Y	Z
C	1.12715	-2.9577	2.851872
C	2.190167	-3.08774	2.033654
C	3.451933	-2.64791	2.58006
C	3.349878	-2.16967	3.838525
S	1.634672	-2.24451	4.425447
C	4.383778	-1.6666	4.736819
C	4.956325	-0.44616	4.803533
C	5.92813	-0.33594	5.874407
C	6.10066	-1.44038	6.616268
S	5.03564	-2.76239	6.031693
C	7.199719	-2.2665	-1.12769
C	7.621238	-1.36155	-2.05627
C	8.735877	-0.52525	-1.82302
C	9.42775	-0.63347	-0.59584
C	8.980829	-1.58757	0.351273
C	7.896113	-2.37878	0.095892
C	9.161504	0.433506	-2.77649
C	10.23577	1.224703	-2.54153
C	10.96273	1.096157	-1.32901
C	10.56131	0.218116	-0.35202
C	8.843882	2.143381	3.075639
C	9.399914	1.536335	1.991623
C	10.62067	0.818802	2.109826
C	11.23662	0.743449	3.378515
C	10.63847	1.405801	4.479989
C	9.474232	2.096266	4.338887
C	11.24053	0.172172	0.985815
C	12.42302	-0.50097	1.170027
C	13.00763	-0.61414	2.456625
C	12.43396	-0.00103	3.521191
C	4.730488	-2.7574	1.787226
O	7.526499	-3.23848	1.10384
O	7.664528	2.848516	2.91519
N	12.06598	1.949906	-1.07247
N	13.01909	-1.16037	0.063673

C	13.08724	2.292658	-1.89463
C	14.31753	-1.14778	-0.32289
N	13.96222	3.143874	-1.33711
S	13.28906	1.684804	-3.51812
N	14.56045	-1.88338	-1.41844
S	15.56914	-0.25063	0.4966
C	15.15014	3.660978	-1.94834
C	15.82226	-2.02805	-2.08181
C	15.09667	4.835937	-2.67181
C	16.24989	5.37267	-3.23084
C	17.45869	4.717305	-3.05441
C	17.51573	3.534955	-2.32438
C	16.36525	3.006868	-1.77454
C	16.65921	-3.08766	-1.75176
C	17.85399	-3.26163	-2.41912
C	18.22063	-2.37049	-3.42241
C	17.39041	-1.3114	-3.75461
C	16.18766	-1.14419	-3.07882
C	6.580351	-4.31849	0.986397
C	6.487148	2.450289	3.652943
C	5.437997	-4.11182	1.969886
C	5.833921	1.212508	3.04777
C	4.628454	0.701903	3.878582
H	2.111856	-3.4917	1.043862
H	6.472492	0.564683	6.071365
H	6.342336	-2.87094	-1.3347
H	7.094372	-1.27054	-2.98733
H	8.61361	0.534091	-3.69389
H	10.55203	1.945196	-3.26131
H	11.12783	1.362046	5.4345
H	9.039758	2.6186	5.167936
H	13.90848	-1.17499	2.568151
H	12.88403	-0.08224	4.492024
H	5.414864	-1.97161	2.071332
H	4.495437	-2.60655	0.73716
H	12.14211	2.23839	-0.12187
H	12.36678	-1.57871	-0.56234
H	13.82827	3.394969	-0.38218

H	13.78913	-2.33238	-1.86139
H	14.15507	5.329419	-2.81103
H	16.18747	6.281578	-3.7927
H	18.46279	3.05029	-2.20465
H	16.398	2.089692	-1.22052
H	16.375	-3.76189	-0.96803
H	18.51742	-4.06725	-2.18051
H	17.65882	-0.61376	-4.52065
H	15.54373	-0.32223	-3.32225
H	7.123224	-5.22391	1.216607
H	6.209523	-4.39881	-0.02494
H	5.828627	3.302597	3.594631
H	6.748382	2.294511	4.691045
H	5.818696	-4.17896	2.981265
H	4.731037	-4.92687	1.839338
H	6.571055	0.425211	2.944531
H	5.50757	1.465264	2.045729
H	4.216679	1.520657	4.465457
H	3.839928	0.39021	3.208376
C	19.94382	-1.75595	-5.04382
H	20.8955	-2.17588	-5.3197
H	19.29242	-1.73117	-5.90765
H	20.08378	-0.75284	-4.66304
C	18.73331	6.355747	-4.34673
H	19.77113	6.455031	-4.61366
H	18.13253	6.274551	-5.24329
H	18.42085	7.219795	-3.77461
O	19.42296	-2.62063	-4.03242
O	18.65151	5.164127	-3.56399
H	8.895809	1.607268	1.051384
H	9.475517	-1.70111	1.292384
C	-11.9246	-8.3616	-3.75837
C	-12.1799	-7.06435	-3.98509
C	-11.326	-6.12581	-3.28268
C	-10.4112	-6.72943	-2.49687
S	-10.557	-8.53861	-2.60635
C	-9.38691	-6.15382	-1.63308
C	-9.50213	-5.57937	-0.41565

C	-8.23084	-5.17392	0.1394
C	-7.15397	-5.4307	-0.62824
S	-7.65446	-6.22385	-2.16475
C	-13.0063	-0.26637	-4.16353
C	-13.5824	0.81302	-3.55971
C	-12.9291	1.518579	-2.52525
C	-11.6284	1.118159	-2.14124
C	-11.0513	-0.01133	-2.77189
C	-11.7338	-0.70105	-3.73492
C	-13.5451	2.613995	-1.8648
C	-12.9041	3.274877	-0.87165
C	-11.5929	2.888672	-0.48849
C	-10.9496	1.850422	-1.10865
C	-9.89078	-1.26317	1.709075
C	-10.2279	-0.28724	0.821714
C	-9.22181	0.516354	0.223218
C	-7.87041	0.285072	0.567962
C	-7.56525	-0.70391	1.536051
C	-8.54689	-1.4593	2.098153
C	-9.5301	1.522503	-0.75119
C	-8.49996	2.193731	-1.35493
C	-7.14824	1.950055	-1.01758
C	-6.84569	1.031323	-0.06763
C	-11.5017	-4.63733	-3.44468
O	-11.1198	-1.82593	-4.23453
O	-10.8867	-2.06053	2.245177
N	-10.8977	3.663567	0.475644
N	-8.7813	3.121574	-2.40826
C	-11.2727	3.942633	1.741857
C	-8.58702	4.45199	-2.38981
N	-10.3964	4.738434	2.384473
S	-12.7265	3.329403	2.476208
N	-8.87195	5.072443	-3.54685
S	-8.02447	5.311567	-0.97359
C	-10.4851	5.192005	3.735426
C	-8.80699	6.48125	-3.79559
C	-10.2519	4.329597	4.79008
C	-10.2922	4.790603	6.099185

C	-10.5572	6.129129	6.345506
C	-10.7853	7.002247	5.289079
C	-10.755	6.531253	3.991504
C	-7.64304	7.045655	-4.30521
C	-7.5939	8.393611	-4.59223
C	-8.71161	9.191109	-4.36718
C	-9.87482	8.635229	-3.85732
C	-9.91451	7.275831	-3.57358
C	-11.7622	-2.75476	-5.1241
C	-10.8153	-3.49004	2.053272
C	-11.1865	-4.13552	-4.86644
C	-11.1352	-3.89009	0.61493
C	-10.8079	-5.37678	0.317351
H	-12.9669	-6.74253	-4.6389
H	-8.14687	-4.70361	1.096668
H	-13.5248	-0.77325	-4.95023
H	-14.5581	1.137206	-3.8691
H	-14.5334	2.910358	-2.16062
H	-13.368	4.08884	-0.35761
H	-6.54051	-0.85481	1.81621
H	-8.31234	-2.19456	2.841818
H	-6.38336	2.512762	-1.51133
H	-5.82382	0.846648	0.202473
H	-10.8814	-4.10495	-2.73947
H	-12.5337	-4.38975	-3.20378
H	-10.0108	4.015158	0.178172
H	-9.21215	2.723156	-3.21412
H	-9.60191	5.057252	1.871694
H	-9.15616	4.509514	-4.31853
H	-10.0555	3.295339	4.592349
H	-10.1189	4.103321	6.901234
H	-10.9909	8.030071	5.507087
H	-10.9463	7.196583	3.172366
H	-6.78074	6.428379	-4.46211
H	-6.70789	8.851608	-4.98081
H	-10.7418	9.235254	-3.67365
H	-10.8067	6.839745	-3.16912
H	-11.588	-2.43594	-6.14414

H	-12.8291	-2.76901	-4.94252
H	-11.5417	-3.89338	2.740763
H	-9.8379	-3.8462	2.351526
H	-10.1146	-4.11998	-5.021
H	-11.6061	-4.81075	-5.60663
H	-10.5864	-3.25214	-0.06678
H	-12.1865	-3.69847	0.435733
H	-10.7796	-5.93953	1.248212
H	-11.6034	-5.81223	-0.26984
C	-9.61662	11.46204	-4.44572
H	-9.22015	12.41224	-4.75791
H	-10.4958	11.22455	-5.03023
H	-9.87331	11.5006	-3.39532
C	-10.4514	5.884341	8.775683
H	-10.5666	6.563242	9.602819
H	-11.2091	5.113469	8.828208
H	-9.46819	5.432991	8.810313
O	-8.57152	10.51575	-4.68318
O	-10.6103	6.681013	7.601249
H	-11.2593	-0.14108	0.57893
H	-10.0876	-0.37164	-2.47748
C	-12.6135	-9.56212	-4.32018
H	-11.9263	-10.1866	-4.87957
H	-13.0491	-10.1719	-3.53693
H	-13.4086	-9.25514	-4.98822
C	7.015192	-1.65004	7.77855
H	7.731833	-2.43875	7.580109
H	7.565776	-0.74101	7.98605
H	6.464515	-1.92158	8.671812
C	-0.25478	-3.30045	2.606109
C	-1.30127	-3.28498	3.457584
S	-0.78771	-3.84415	0.965768
C	-2.54672	-3.69684	2.881259
H	-1.20886	-2.99691	4.484654
C	-2.4783	-4.03532	1.576429
H	-3.45514	-3.742	3.446287
C	-3.52539	-4.4943	0.695773
C	-3.435	-4.97018	-0.56387

S	-5.24919	-4.48067	1.239479
C	-4.68689	-5.34255	-1.15294
H	-2.50312	-5.06894	-1.08189
C	-5.76082	-5.15929	-0.35702
H	-4.76201	-5.73549	-2.14602

Total energy = -9227.447478 Hartree

PT1$_{5\text{-mer}}$ + isophthalate

Symbol	X	Y	Z
C	3.035864	-3.9946	-3.06686
C	2.193864	-3.36356	-2.22812
C	0.799787	-3.39121	-2.61524
C	0.583073	-4.03626	-3.78166
S	2.137684	-4.67529	-4.47597
C	-0.65912	-4.2754	-4.50531
C	-1.47214	-3.41215	-5.15089
C	-2.61174	-4.0631	-5.76981
C	-2.67749	-5.39294	-5.61202
S	-1.28432	-5.97896	-4.6437
C	-2.81711	-0.21838	1.183654
C	-3.59953	0.885087	1.34713
C	-4.872	0.973241	0.731852
C	-5.35051	-0.11757	-0.03382
C	-4.51561	-1.251	-0.1938
C	-3.27157	-1.28704	0.378085
C	-5.66343	2.140177	0.850281
C	-6.8849	2.227582	0.262095
C	-7.39446	1.121445	-0.45611
C	-6.65797	-0.02782	-0.62442
C	-5.47918	-0.75449	-4.70847
C	-5.84644	-0.53402	-3.41648
C	-6.80173	-1.37552	-2.78266
C	-7.34813	-2.45038	-3.52027
C	-6.97326	-2.62133	-4.8765
C	-6.06892	-1.79294	-5.46484
C	-7.2085	-1.17714	-1.4206
C	-8.09865	-2.06	-0.85529
C	-8.5922	-3.17182	-1.58131
C	-8.23996	-3.34408	-2.87978
C	-0.25422	-2.75658	-1.74359
O	-2.50837	-2.39164	0.105597
O	-4.53087	0.067433	-5.28858
N	-8.66132	1.210058	-1.10434
N	-8.44585	-1.87939	0.509315

C	-9.84237	1.650147	-0.63101
C	-9.63724	-2.0092	1.12891
N	-10.844	1.564978	-1.53133
S	-10.1056	2.267716	0.979358
N	-9.58952	-1.71886	2.441361
S	-11.1261	-2.49152	0.347537
C	-12.2117	1.918742	-1.31393
C	-10.6923	-1.7203	3.35255
C	-12.6623	3.175087	-1.66758
C	-14.0009	3.515872	-1.51139
C	-14.8823	2.580637	-0.99376
C	-14.433	1.314922	-0.63456
C	-13.1019	0.985182	-0.79249
C	-10.9463	-2.84384	4.129831
C	-11.9742	-2.83417	5.051347
C	-12.7572	-1.69514	5.197443
C	-12.5104	-0.57186	4.424449
C	-11.473	-0.58971	3.500096
C	-1.16573	-2.58439	0.611155
C	-3.30964	-0.51842	-5.80127
C	-0.41359	-3.45247	-0.37824
C	-2.41172	-1.06546	-4.69463
C	-1.24252	-1.92126	-5.24961
H	2.521512	-2.88844	-1.32341
H	-3.35772	-3.5232	-6.31609
H	-1.86548	-0.24976	1.668155
H	-3.22723	1.693712	1.949422
H	-5.26994	2.97133	1.402827
H	-7.47408	3.11339	0.345071
H	-7.41811	-3.42236	-5.43673
H	-5.8011	-1.9138	-6.49574
H	-9.25151	-3.85674	-1.09841
H	-8.62493	-4.18034	-3.43206
H	-1.20867	-2.7481	-2.24774
H	0.027347	-1.72053	-1.57418
H	-8.67125	0.797542	-2.01077
H	-7.70504	-1.4874	1.049218
H	-10.641	1.155377	-2.41628

H	-8.72772	-1.38364	2.813407
H	-11.9676	3.894856	-2.05352
H	-14.3285	4.497108	-1.78698
H	-15.1354	0.614628	-0.23122
H	-12.7414	0.017978	-0.50202
H	-10.3424	-3.72039	4.001889
H	-12.1895	-3.68848	5.659865
H	-13.1055	0.312521	4.521537
H	-11.2785	0.269413	2.889188
H	-1.2277	-3.04898	1.58545
H	-0.66062	-1.63711	0.715138
H	-2.82846	0.289832	-6.32865
H	-3.55988	-1.28767	-6.52085
H	-0.92727	-4.39847	-0.50711
H	0.558088	-3.65855	0.054338
H	-3.00035	-1.65695	-4.00492
H	-2.02297	-0.22987	-4.12582
H	-1.06407	-1.66227	-6.2922
H	-0.33272	-1.67438	-4.72311
C	-14.6406	-0.6755	6.371938
H	-15.3178	-1.00752	7.140468
H	-14.0989	0.195972	6.716384
H	-15.1979	-0.4264	5.478124
C	-16.7957	4.09805	-1.06919
H	-17.8362	4.011247	-0.80633
H	-16.3307	4.866959	-0.46557
H	-16.7069	4.35619	-2.11694
O	-13.7575	-1.7715	6.138639
O	-16.224	2.819204	-0.80313
H	-5.38123	0.264073	-2.87736
H	-4.82496	-2.08688	-0.7856
C	12.95579	-4.85394	2.58668
C	12.0605	-4.1004	3.242398
C	10.97319	-3.577	2.438512
C	11.05266	-3.94017	1.141967
S	12.5134	-4.98183	0.847341
C	10.11915	-3.63459	0.064638
C	9.949529	-2.48805	-0.62904

C	8.815702	-2.53698	-1.52921
C	8.141551	-3.70037	-1.53932
S	8.888752	-4.8772	-0.40114
C	6.829965	0.09906	5.041944
C	6.482704	1.408144	5.210642
C	5.995638	2.185481	4.139833
C	5.772388	1.565562	2.888421
C	6.131809	0.204263	2.73442
C	6.693574	-0.48998	3.769494
C	5.76142	3.578632	4.282653
C	5.406581	4.323658	3.212713
C	5.192741	3.718503	1.944499
C	5.281991	2.358858	1.790581
C	7.889787	1.842063	-1.63662
C	7.079278	2.000451	-0.55505
C	5.698351	1.674792	-0.64182
C	5.192416	1.205248	-1.87463
C	6.052769	1.134318	-2.99773
C	7.374618	1.438366	-2.88634
C	4.836762	1.717695	0.50686
C	3.572429	1.193488	0.402446
C	3.065894	0.76064	-0.84873
C	3.840753	0.786992	-1.95793
C	9.88191	-2.72031	3.03262
O	7.15739	-1.76403	3.483563
O	9.260346	2.063688	-1.52137
N	4.848127	4.572053	0.857626
N	2.704638	1.105338	1.531881
C	5.691105	5.46806	0.285881
C	2.284321	-0.04321	2.106502
N	5.118705	6.176887	-0.68931
S	7.354829	5.654518	0.845341
N	1.447039	0.148552	3.128605
S	2.777357	-1.62879	1.49921
C	5.639326	7.176599	-1.54186
C	0.828293	-0.77457	4.00368
C	6.935461	7.196559	-2.03088
C	7.342586	8.195819	-2.91096

C	6.446742	9.165769	-3.31941
C	5.138636	9.137289	-2.85683
C	4.735465	8.153336	-1.9761
C	1.447295	-1.92083	4.501231
C	0.784751	-2.73382	5.402235
C	-0.4919	-2.40802	5.836186
C	-1.10513	-1.2587	5.369994
C	-0.44429	-0.44522	4.456575
C	7.785804	-2.61485	4.444856
C	10.12029	0.913385	-1.59643
C	8.75226	-3.52443	3.702167
C	10.07868	0.068034	-0.3258
C	10.83796	-1.27231	-0.49207
H	12.14185	-3.8936	4.292136
H	8.506214	-1.70071	-2.12235
H	7.202596	-0.46041	5.87467
H	6.600476	1.871625	6.172535
H	5.905908	4.036173	5.243245
H	5.278497	5.382854	3.288422
H	5.645506	0.818008	-3.93947
H	8.030706	1.388257	-3.7337
H	2.053551	0.416706	-0.88752
H	3.450482	0.466478	-2.90516
H	9.441929	-2.09252	2.273224
H	10.32966	-2.0566	3.768689
H	3.896744	4.582465	0.528173
H	2.313402	1.954833	1.899573
H	4.108865	6.037808	-0.78192
H	1.109261	1.109358	3.235294
H	7.632959	6.449481	-1.7242
H	8.354718	8.187733	-3.26286
H	4.451326	9.886653	-3.19436
H	3.715644	8.123002	-1.63638
H	2.431854	-2.17643	4.177415
H	1.248007	-3.62112	5.784876
H	-2.08399	-0.97329	5.698127
H	-0.91477	0.458243	4.111197
H	7.026232	-3.18666	4.964251

H	8.333857	-2.03075	5.173761
H	11.11025	1.313502	-1.75854
H	9.85275	0.316678	-2.46231
H	8.211541	-4.0876	2.952306
H	9.166368	-4.23747	4.411742
H	9.049252	-0.11777	-0.04837
H	10.51045	0.645734	0.482558
H	11.47995	-1.21844	-1.37055
H	11.50163	-1.42632	0.347036
C	-2.38263	-3.04415	7.240971
H	-2.59664	-3.86334	7.907895
H	-2.43205	-2.11022	7.78732
H	-3.11245	-3.02878	6.440808
C	8.078352	10.30795	-4.72856
H	8.065022	11.17877	-5.36421
H	8.814848	10.44376	-3.94544
H	8.341345	9.436432	-5.3164
O	-1.07241	-3.28235	6.74101
O	6.767764	10.19603	-4.19347
H	7.494957	2.363514	0.361217
H	5.991636	-0.29535	1.799121
C	14.17716	-5.52911	3.119711
H	14.1321	-6.60296	2.974789
H	15.07599	-5.16876	2.631305
H	14.27267	-5.33741	4.18172
C	-3.71626	-6.33997	-6.11715
H	-4.21154	-6.85483	-5.30144
H	-4.4693	-5.79962	-6.67819
H	-3.28539	-7.09247	-6.76863
C	4.464476	-4.18592	-2.92855
C	5.245352	-5.1727	-3.4215
S	5.433422	-3.0491	-1.92604
C	6.619461	-5.0953	-3.01551
H	4.862088	-5.96248	-4.03506
C	6.903017	-4.04399	-2.22036
H	7.356143	-5.81816	-3.30297
C	-0.68267	4.372636	1.896981
C	0.39779	4.82116	1.152268

C	0.30863	5.958318	0.364955
C	-0.88462	6.669925	0.337284
C	-1.97427	6.232039	1.078989
C	-1.87678	5.082989	1.852312
H	1.316273	4.280973	1.181734
H	-0.93867	7.554818	-0.26526
H	-2.89614	6.784017	1.05379
H	-2.70984	4.730935	2.42855
C	1.502345	6.409683	-0.43389
O	1.443691	7.505556	-1.03262
C	-0.54691	3.122267	2.716255
O	-1.58847	2.574373	3.16447
O	0.636746	2.679747	2.889226
O	2.510482	5.620446	-0.43218

Total energy = -9282.018466 Hartree

PT2$_{6\text{-mer}}$ + isophthalate

Symbol	X	Y	Z
C	-1.04644	5.225229	2.220678
C	-0.32395	4.13274	1.910608
C	1.108152	4.269502	2.046745
C	1.482561	5.497059	2.466979
S	0.035226	6.566711	2.744426
C	2.813332	6.040287	2.709985
C	3.774588	6.405884	1.835511
C	4.960086	6.932352	2.485183
C	4.915996	6.974898	3.825024
S	3.346642	6.341507	4.421971
C	3.98039	-0.53914	-0.33775
C	4.763741	-0.98045	-1.36095
C	6.133324	-0.63412	-1.46145
C	6.71428	0.16558	-0.44904
C	5.886209	0.6267	0.604932
C	4.55586	0.304793	0.643574
C	6.92873	-1.05178	-2.55379
C	8.244374	-0.72394	-2.63899
C	8.849287	0.021324	-1.59987
C	8.113658	0.47984	-0.53071
C	7.753842	4.777746	-0.40375
C	7.861718	3.421316	-0.43557
C	8.626837	2.73409	0.545826
C	9.258936	3.488938	1.559949
C	9.156315	4.902338	1.535919
C	8.431023	5.537907	0.576383
C	8.759432	1.304349	0.546494
C	9.473037	0.703876	1.554758
C	10.054	1.462069	2.60063
C	9.962882	2.81497	2.588107
C	2.029576	3.132979	1.677107
O	3.829626	0.859837	1.662538
O	6.985195	5.414187	-1.36196
N	10.22242	0.392384	-1.67682
N	9.55042	-0.7142	1.578089

C	11.30249	-0.31289	-2.06837
C	10.62939	-1.50225	1.765216
N	12.45477	0.381515	-1.96439
S	11.28055	-1.95872	-2.64911
N	10.35704	-2.81568	1.685756
S	12.24343	-0.90388	2.072196
C	13.76386	-0.09597	-2.28353
C	11.30202	-3.88639	1.777547
C	14.28304	0.110277	-3.54629
C	15.57377	-0.30209	-3.85609
C	16.33863	-0.92401	-2.88247
C	15.82047	-1.13383	-1.60967
C	14.53641	-0.72374	-1.31161
C	11.49	-4.54266	2.987879
C	12.36003	-5.61119	3.070929
C	13.05113	-6.02636	1.938587
C	12.86955	-5.37471	0.729052
C	11.98993	-4.30186	0.653396
C	2.410957	0.632201	1.85658
C	5.869696	6.233749	-0.93813
C	1.840693	1.865774	2.533117
C	4.737586	5.411736	-0.32887
C	3.650729	6.298426	0.333093
H	-0.77608	3.230485	1.550984
H	5.818558	7.265947	1.938987
H	2.950244	-0.8408	-0.29812
H	4.330007	-1.60889	-2.1155
H	6.470569	-1.62753	-3.33523
H	8.833441	-1.03556	-3.47124
H	9.664179	5.468574	2.293942
H	8.370616	6.607815	0.551666
H	10.57272	0.953713	3.382506
H	10.41311	3.390528	3.37441
H	3.060064	3.447852	1.741075
H	1.842326	2.87994	0.635867
H	10.40448	1.291819	-1.29006
H	8.706775	-1.16328	1.294357
H	12.41938	1.280657	-1.53715

H	9.420961	-3.08644	1.475886
H	13.67682	0.581644	-4.29448
H	15.95499	-0.13798	-4.84293
H	16.43236	-1.62183	-0.87904
H	14.11987	-0.89613	-0.33889
H	10.95966	-4.20809	3.857554
H	12.52142	-6.13204	3.992343
H	13.3959	-5.6788	-0.15191
H	11.84947	-3.78459	-0.27523
H	2.287643	-0.2485	2.470531
H	1.922188	0.453225	0.912011
H	5.547219	6.73088	-1.83944
H	6.224433	6.987776	-0.24747
H	2.306529	2.007956	3.501404
H	0.786454	1.677949	2.710792
H	5.138358	4.720767	0.401815
H	4.296085	4.806792	-1.11147
H	3.684876	7.298909	-0.09571
H	2.673207	5.905809	0.094755
C	14.66632	-7.61679	1.028091
H	15.22981	-8.43619	1.440712
H	14.02716	-7.97872	0.233116
H	15.34391	-6.86879	0.637203
C	18.24817	-1.26954	-4.36725
H	19.22285	-1.71149	-4.24932
H	17.68868	-1.81633	-5.11547
H	18.35333	-0.23693	-4.67544
O	13.89602	-7.09712	2.111103
O	17.62564	-1.36478	-3.088
H	7.33768	2.879201	-1.19413
H	6.276202	1.255186	1.378047
C	-15.3959	1.310208	-2.1788
C	-14.7362	0.149472	-2.05195
C	-13.3126	0.262151	-1.79737
C	-12.8839	1.539	-1.73747
S	-14.266	2.69695	-1.99062
C	-11.5402	2.050906	-1.48954
C	-10.5004	2.184079	-2.34213

C	-9.30684	2.689332	-1.70144
C	-9.41602	2.920551	-0.38099
S	-11.0843	2.562779	0.188443
C	-9.60697	-4.759	-0.94573
C	-8.53805	-5.47345	-1.40625
C	-7.27038	-4.87661	-1.57458
C	-7.09524	-3.52581	-1.19517
C	-8.21183	-2.8031	-0.71454
C	-9.43787	-3.39723	-0.62736
C	-6.17523	-5.59039	-2.13001
C	-4.9921	-4.97269	-2.35114
C	-4.81131	-3.60922	-1.99661
C	-5.81211	-2.90679	-1.37559
C	-6.30665	0.648496	-3.81155
C	-6.19489	-0.46876	-3.04275
C	-5.55225	-0.40296	-1.77502
C	-5.02825	0.835131	-1.34767
C	-5.06074	1.943979	-2.22922
C	-5.68954	1.860519	-3.43332
C	-5.52792	-1.52208	-0.87616
C	-5.16397	-1.31719	0.4309
C	-4.62436	-0.07293	0.842231
C	-4.51765	0.955756	-0.0317
C	-12.4335	-0.95058	-1.63254
O	-10.4893	-2.58733	-0.23862
O	-7.06936	0.622616	-4.97955
N	-3.547	-3.0019	-2.25285
N	-5.2656	-2.35964	1.397584
C	-3.02594	-2.74481	-3.4777
C	-6.13693	-2.37963	2.43309
N	-1.85307	-2.10366	-3.41675
S	-3.85457	-3.24953	-4.94817
N	-6.03804	-3.48133	3.180417
S	-7.24949	-1.04426	2.7449
C	-0.96916	-1.62162	-4.40809
C	-6.74354	-3.89039	4.336145
C	-1.28008	-1.39147	-5.73922
C	-0.32035	-0.87882	-6.61064

C	0.947986	-0.58041	-6.15528
C	1.263284	-0.78694	-4.81886
C	0.319281	-1.30041	-3.95546
C	-8.10483	-3.67515	4.544389
C	-8.72256	-4.17112	5.678364
C	-7.99999	-4.90321	6.607656
C	-6.65366	-5.1449	6.398408
C	-6.02902	-4.6382	5.26534
C	-11.854	-3.00169	-0.27781
C	-8.27035	1.420029	-4.98382
C	-12.6999	-1.74026	-0.33965
C	-9.40269	0.779365	-4.17893
C	-10.5208	1.790647	-3.8018
H	-15.2265	-0.80126	-2.13531
H	-8.37577	2.809375	-2.21538
H	-10.558	-5.23705	-0.82562
H	-8.65798	-6.51057	-1.65914
H	-6.30522	-6.62424	-2.38946
H	-4.16979	-5.48972	-2.7978
H	-4.60318	2.863746	-1.91811
H	-5.72258	2.699278	-4.10132
H	-4.29569	0.021801	1.856122
H	-4.08022	1.885162	0.270978
H	-11.3946	-0.65938	-1.64126
H	-12.5867	-1.60817	-2.48657
H	-2.9961	-2.71292	-1.46429
H	-4.61348	-3.12549	1.355481
H	-1.49342	-1.96216	-2.47439
H	-5.25079	-4.08809	2.943583
H	-2.25549	-1.61179	-6.10661
H	-0.59605	-0.71968	-7.63393
H	2.245797	-0.53663	-4.4721
H	0.570814	-1.4398	-2.92047
H	-8.66949	-3.11639	3.831548
H	-9.76682	-4.00402	5.852232
H	-6.07256	-5.7193	7.091104
H	-4.9874	-4.84557	5.09707
H	-12.0835	-3.58653	0.605446

H	-12.0381	-3.61534	-1.15365
H	-8.53956	1.517916	-6.02499
H	-8.04458	2.408762	-4.60172
H	-12.4719	-1.12028	0.518486
H	-13.7485	-2.02103	-0.27217
H	-8.99081	0.339958	-3.28017
H	-9.81822	-0.03994	-4.75379
H	-10.424	2.688044	-4.41057
H	-11.4906	1.374129	-4.03559
C	-8.05138	-6.11085	8.726451
H	-8.8068	-6.33292	9.462971
H	-7.64434	-7.0357	8.336011
H	-7.25527	-5.53793	9.186225
C	1.73286	0.197002	-8.33026
H	2.662406	0.585367	-8.7138
H	1.468966	-0.70571	-8.86833
H	0.950061	0.93387	-8.46554
O	-8.70637	-5.36336	7.710211
O	1.960761	-0.06906	-6.95346
H	-6.6262	-1.38584	-3.38532
H	-8.11444	-1.78049	-0.41618
C	-16.8496	1.533623	-2.44044
H	-17.315	2.09516	-1.63776
H	-17.008	2.084151	-3.36142
H	-17.3616	0.582556	-2.52541
C	5.964557	7.451687	4.776068
H	6.28417	6.65769	5.441655
H	6.832223	7.799427	4.228327
H	5.602589	8.269634	5.38921
C	-2.48184	5.38753	2.113221
C	-3.21257	6.474457	1.787419
S	-3.54374	3.945173	2.291481
C	-4.61673	6.217173	1.620429
H	-2.77372	7.436925	1.61837
C	-4.97835	4.932357	1.820631
H	-5.31659	6.974067	1.329384
C	-1.3852	-4.29785	2.690763
C	-1.29734	-3.53401	1.536913

C	-0.08752	-3.00654	1.11054
C	1.062448	-3.27551	1.844936
C	0.990095	-4.04975	2.995136
C	-0.23161	-4.55277	3.4224
H	-2.17855	-3.34228	0.968943
H	1.999271	-2.88187	1.503729
H	1.881915	-4.2572	3.557929
H	-0.31281	-5.13834	4.316432
C	-0.03979	-2.16043	-0.13176
O	1.047932	-1.59599	-0.43121
C	-2.72469	-4.80583	3.15722
O	-2.79001	-5.38174	4.26582
O	-3.71694	-4.57738	2.381706
O	-1.11395	-2.06808	-0.80517
C	-6.24767	4.26518	1.604468
C	-6.76422	3.199662	2.248254
S	-7.27063	4.699127	0.176466
C	-7.95399	2.659183	1.653188
H	-6.30055	2.74896	3.101761
C	-8.35927	3.301383	0.541961
H	-8.42059	1.766208	2.013394

Total energy = -9832.058698 Hartree

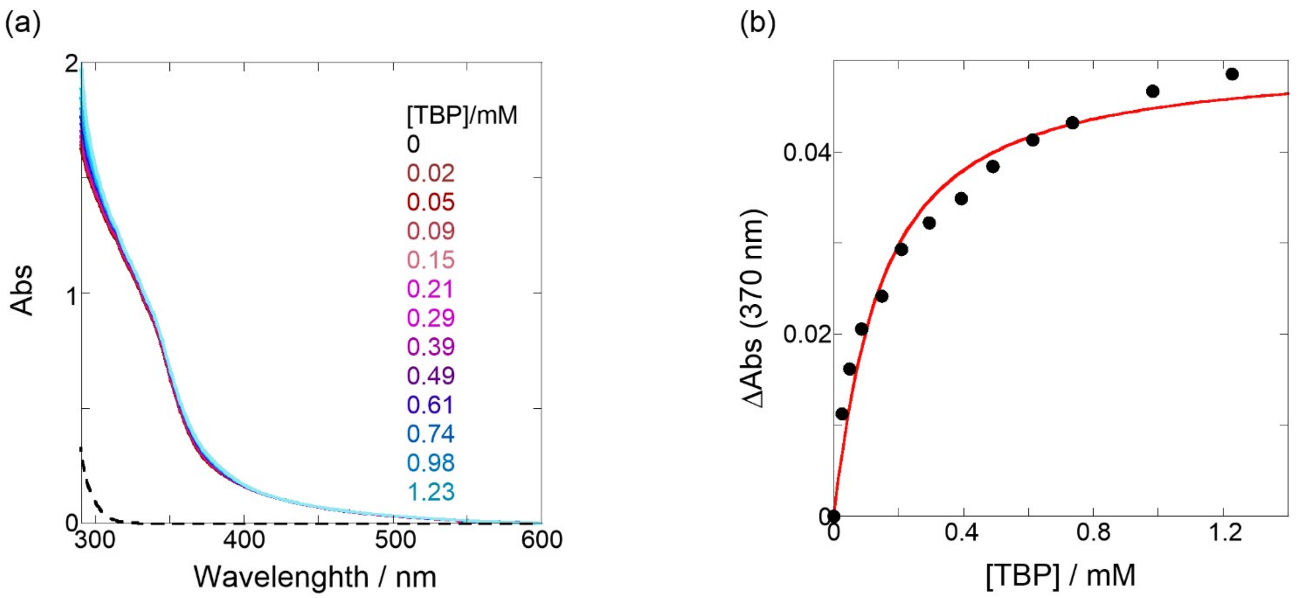

Figure S10. (a) UV/vis spectral changes of a chloroform solution of **PT1** (45.8 μM, black) upon gradual addition of TBP (0.02-1.23 mM, colored lines); the dotted line represents the UV spectrum of TBP only. (b) Nonlinear least-squares fitting, assuming 1:1 model, to determine the binding constant.

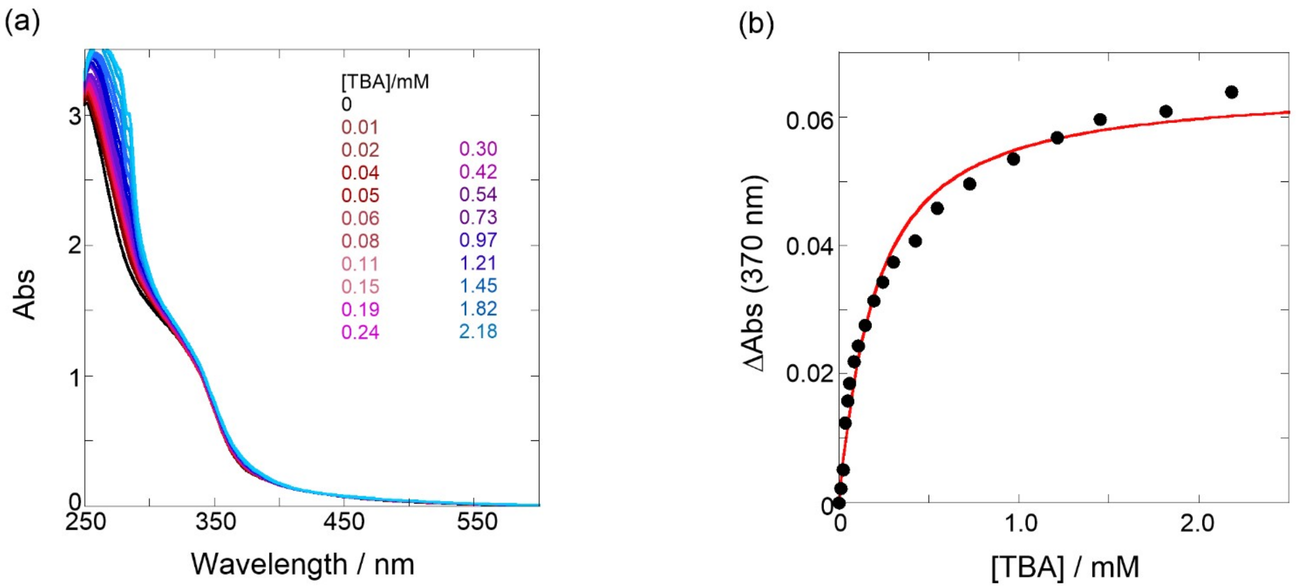

Figure S11. (a) UV/vis spectral changes of a chloroform solution of **PT2** (51.5 μM, black) upon gradual addition of TBA (0.01-2.18 mM, colored lines). (b) Nonlinear least-squares fitting, assuming 1:1 model, to determine the binding constant.

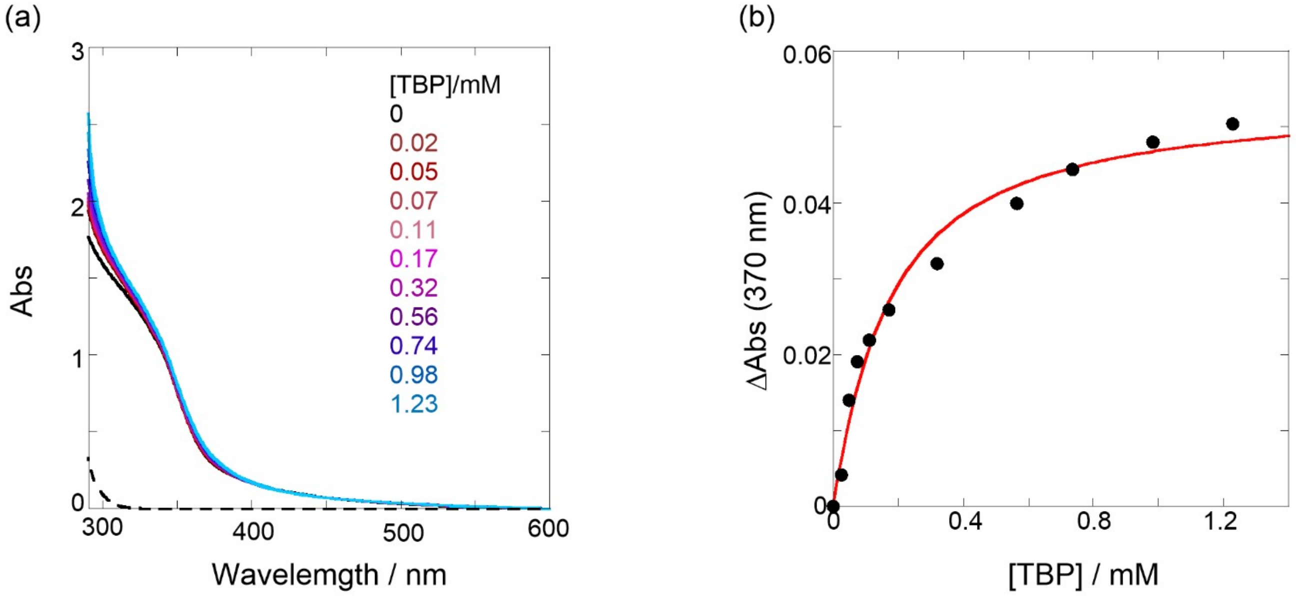

Figure S12. (a) UV/vis spectral changes of a chloroform solution of **PT2** (51.5 μM, black) upon gradual addition of TBP (0.02-1.23 mM, colored lines); the dotted line represents the UV spectrum of TBP only. (b) Nonlinear least-squares fitting, assuming 1:1 model, to determine the binding constant.

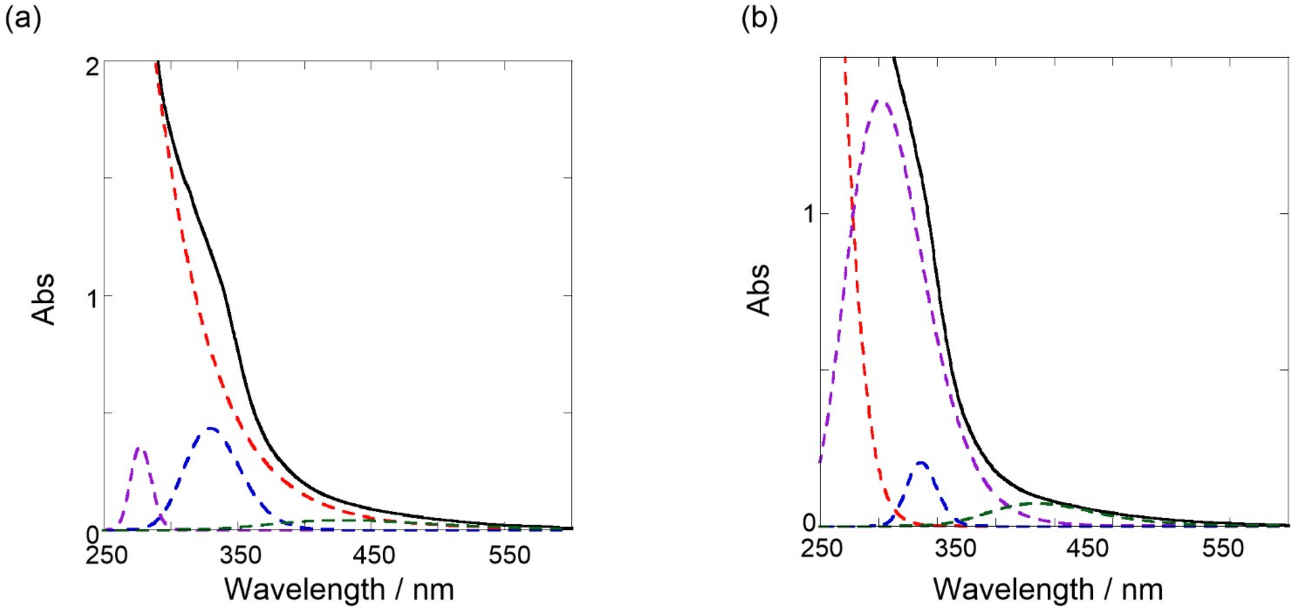

Figure S13. UV/vis spectra (black solid lines) of (a) **PT1** (52.4 μM) with TBA (1.25 mM) and (b) **PT2** (51.5 μM) with TBA (2.18 mM) and results of waveform separation (colored dotted lines).
